# Genome-wide transcript and protein analysis highlights the role of protein homeostasis in the aging mouse heart

**DOI:** 10.1101/gr.275672.121

**Published:** 2022-05

**Authors:** Isabela Gerdes Gyuricza, Joel M. Chick, Gregory R. Keele, Andrew G. Deighan, Steven C. Munger, Ron Korstanje, Steven P. Gygi, Gary A. Churchill

**Affiliations:** 1 The Jackson Laboratory, Bar Harbor, Maine 04609, USA;; 2 Vividion Therapeutics, San Diego, California 92121, USA;; 3 Harvard Medical School, Boston, Massachusetts 02115, USA

## Abstract

Investigation of the molecular mechanisms of aging in the human heart is challenging because of confounding factors, such as diet and medications, as well as limited access to tissues from healthy aging individuals. The laboratory mouse provides an ideal model to study aging in healthy individuals in a controlled environment. However, previous mouse studies have examined only a narrow range of the genetic variation that shapes individual differences during aging. Here, we analyze transcriptome and proteome data from 185 genetically diverse male and female mice at ages 6, 12, and 18 mo to characterize molecular changes that occur in the aging heart. Transcripts and proteins reveal activation of pathways related to exocytosis and cellular transport with age, whereas processes involved in protein folding decrease with age. Additional changes are apparent only in the protein data including reduced fatty acid oxidation and increased autophagy. For proteins that form complexes, we see a decline in correlation between their component subunits with age, suggesting age-related loss of stoichiometry. The most affected complexes are themselves involved in protein homeostasis, which potentially contributes to a cycle of progressive breakdown in protein quality control with age. Our findings highlight the important role of post-transcriptional regulation in aging. In addition, we identify genetic loci that modulate age-related changes in protein homeostasis, suggesting that genetic variation can alter the molecular aging process.

Cardiovascular (CV) diseases are the leading cause of death in elderly people. Improved understanding of mechanisms that underlie the changes that occur in the aging heart could open new opportunities for prevention and treatment (Chiao and Rabinovitch 2015). As the heart ages, characteristic physiological changes occur, including increased arterial thickening and stiffness, endothelium dysfunction, valvular fibrosis and calcification, and a switch from fatty acid to glucose metabolism (Stanley et al. 2005; North and Sinclair 2012; Quarles et al. 2015). Compensatory mechanisms may temporarily maintain heart function but can also contribute to progressive deterioration and eventual heart failure (North and Sinclair 2012). For example, thickening of the left ventricle and remodeling of the extracellular matrix may compensate for loss of systolic function (North and Sinclair 2012; Quarles et al. 2015). However, in the long term, the increased wall stress causes the left ventricle to dilate, leading to a decline in systolic function (Dai et al. 2012). Physiological measures of cardiac function that change with age have high heritability suggesting that genetic factors contribute to variability in cardiac aging in humans (Melzer et al. 2007).

Despite well-known physiological changes in the aging heart, dissecting the cellular and molecular basis of age-related change is challenging because of the complex dynamics and inter-individual variability of the aging process (López-Otín et al. 2013; Singh et al. 2019). Age-related changes at the cellular levels have been associated with loss of protein homeostasis and increased inflammation (López-Otín et al. 2013). Variability of transcript expression increases with age in mammalian tissues, including the heart (Bahar et al. 2006; Işıldak et al. 2020). Age-related dysregulation of transcripts is offset by selective translation, and post-transcriptional mechanisms become crucial for achieving cellular homeostasis (Gonskikh and Polacek 2017). The investigation of molecular mechanisms involved in aging is further complicated by discordant age-related changes between transcripts and their corresponding proteins (Takemon et al. 2021). Waldera-Lupa et al. (2014) found that 77% of the proteins that change with age in human fibroblasts showed no corresponding change in their transcripts (Waldera-Lupa et al. 2014). Thus, investigating age-related changes using only transcriptional profiling may fail to reveal important influences on proteins and higher-order cellular processes.

Mouse models of aging can recapitulate many of the cardiac aging phenotypes seen in humans, such as increased atrial and ventricular dimensions and reduced diastolic function (Lakatta and Levy 2003), and thus provide relevant models for investigating aging processes in the heart. However, most previous studies have used mice descended from only a few isogenic strains that may not reflect the diversity of cardiac phenotypes found in aging human populations. Multiple studies report differences in mouse cardiac phenotypes, under either physiological or pathological conditions, associated with genetic background across inbred strains (Barrick et al. 2007; Xing et al. 2009; Kiper et al. 2013; Avila et al. 2017; Forte et al. 2020), and in multiparent populations (Shorter et al. 2018; Salimova et al. 2019), confirming the importance of genetic diversity in shaping the rate and course of cardiac aging.

In this study we use diversity outbred (DO) mice derived from eight inbred founder strains: A/J (AJ), C57BL/6J (B6), 129S1Sv/ImJ (129), NOD/ShiLtJ (NOD), NZO/H1LtJ (NZO), CAST/EiJ (CAST), PWK/PhJ (PWK), and WSB/EiJ (WSB), to investigate cardiac aging in a genetically and phenotypically diverse model (Svenson et al. 2012; Saul et al. 2019). Aging studies with DO mice can reveal broad patterns of age-related change that occur across different genetic backgrounds and can associate heterogeneity in aging traits to genetic loci. We analyze transcriptome data from RNA sequencing (RNA-seq) and proteome data from mass spectrometry of heart tissues collected from healthy DO mice at ages 6, 12, and 18 mo. At 6 mo of age, the mice have reached full maturity. At 18 mo, most mice are healthy and are only beginning to show signs of age-related decline. Thus, we are looking at changes in transcripts and proteins that are not influenced by developmental programs and are also not reflecting late-stage disease progression (Cellerino and Ori 2017). To characterize molecular and cellular changes in the aging mouse heart, we first identify the transcripts and proteins that change with age and characterize these genes using gene-set enrichment analysis (Subramanian et al. 2005; Yu et al. 2012). We then examine change with age in coregulation of proteins that form multiprotein complexes. Finally, we investigate how genetic variation modulates age-related changes in the heart. The molecular profiling data from this study are freely available to support further investigations of the molecular basis of aging in the mammalian heart (https://qtlviewer.jax.org/viewer/agingheart).

## Results

### Transcripts and proteins reveal age-related changes in immune response, intracellular transport, and protein folding pathways

**Figure 1.**
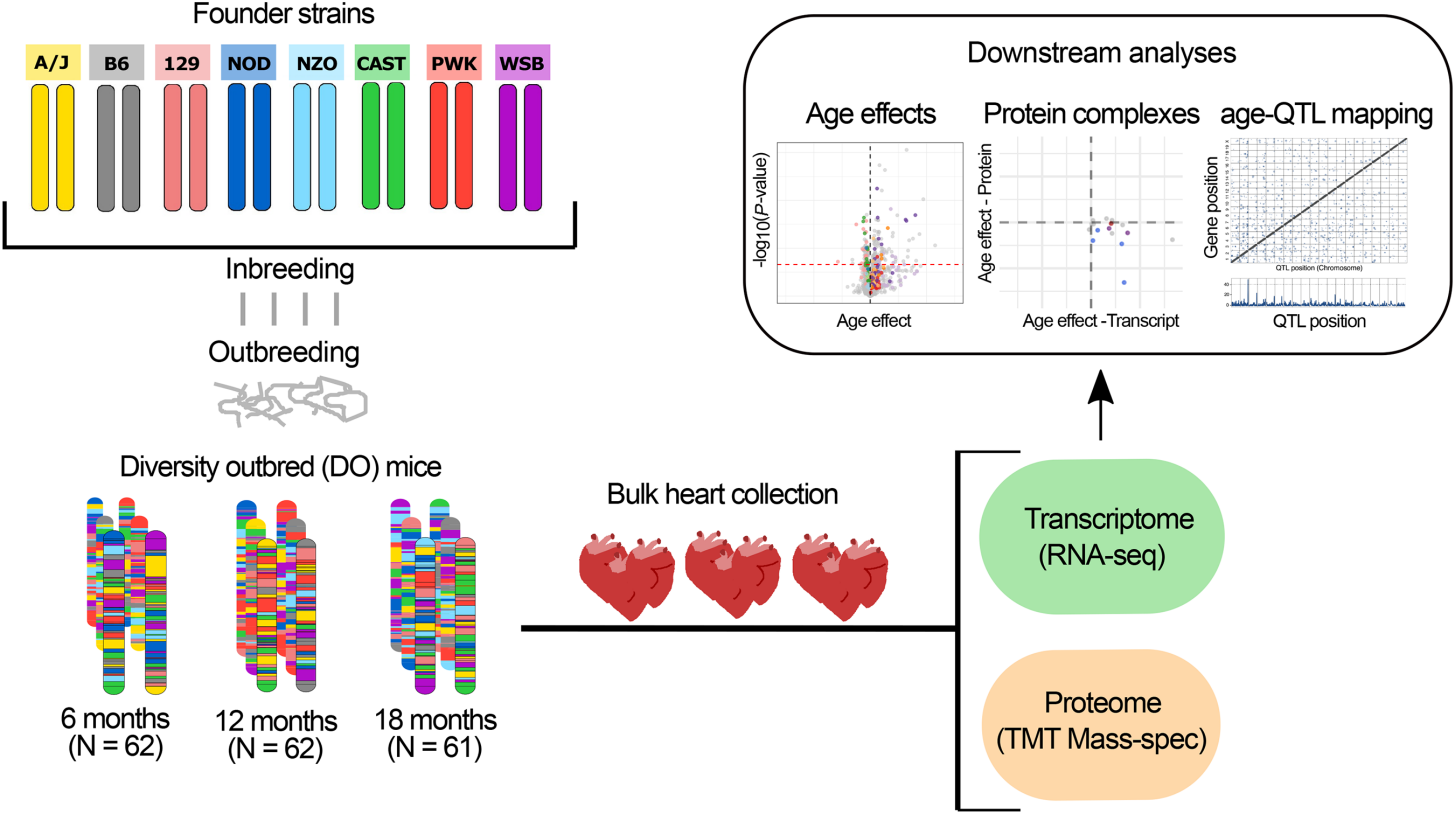
Transcriptome and proteome profiling of the aging heart in genetically diverse mice. DO mice are descended from eight inbred founder strains. Each DO mouse is genetically unique. We obtained cross-sectional samples of tissues from approximately equal numbers of mice aged to 6, 12, or 18 mo. Bulk heart tissue was collected for RNA-seq and mass spectrometry protein analysis. We performed analyses to detect and characterize age-related changes in transcript and protein abundance. We performed functional enrichment of gene sets with age effects, characterized changes in correlation of subunits within protein complexes, and mapped genetic variation that influences age-related changes in transcripts and proteins (age-QTL).

We analyzed heart tissue from 185 DO mice of both sexes (91 females and 94 males) aged to 6, 12, or 18 mo to identify transcripts and proteins that change with age (Fig. 1). We quantified 21,016 unique transcripts after filtering out low expressed genes, and 4221 proteins corresponding to 4151 unique Ensembl gene IDs (release 84). To compare age-related changes across transcripts and proteins, we restricted our analysis to the transcripts for which we have protein data and vice versa. These data consist of 4047 transcripts and 4117 proteins. Transcript expression was summed across all isoforms for a given gene, and some transcripts correspond to two or more proteins. Transcripts and proteins that change with age are referred to throughout as age-related transcripts/proteins, and their magnitude and direction of change are referred to as age effects, reported in units of log_2_ fold change per year, or as standardized age effects (age effect/standard error).

To identify age-related transcripts, we used DESeq2 software (Love et al. 2014) to compute the likelihood ratio test for a linear trend with age, using sex as a covariate (Methods). We identified 206 transcripts with significant age-related changes (false discovery rate [FDR] < 0.01) (Table 1; Supplemental Data S1). To evaluate age-related changes in proteins, we fit a linear regression model to log-scale protein abundance with age as a linear term and sex as a covariate (Methods). We identified 2084 age-related proteins (FDR < 0.01) (Table 1; Supplemental Data S2). We note that 1691 of the age-related proteins are increasing and 393 are decreasing with age. The proportion of proteins that are increasing with age holds across different significance thresholds at about 4:1. For transcripts, the direction of change is roughly balanced, with 122 increasing and 84 decreasing with age (FDR < 0.01). We provide results for the full sets of transcripts and proteins in Supplemental Data S3 and S4.

**Table 1.**
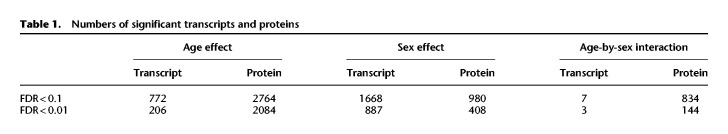
Numbers of significant transcripts and proteins

**Figure 2.**
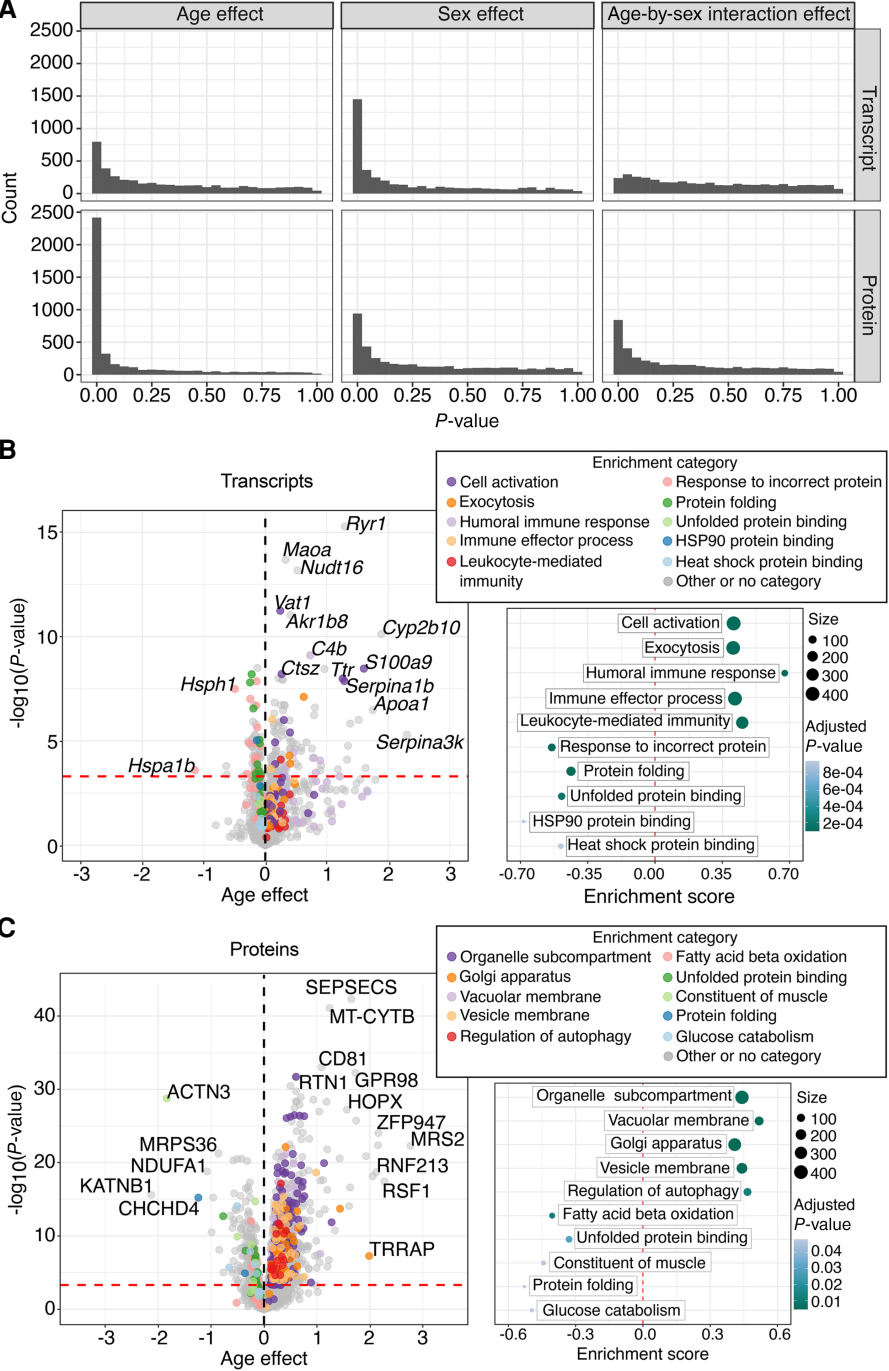
Age-related changes in transcripts and proteins. (*A*) Distribution of *P*-values for tests of (*left* to *right*) age, sex, and age-by-sex interaction effects for transcripts (*top*) and proteins (*bottom*). Volcano plots show the age effects (*x*-axis) and the −log_10_(*P*-values) (*y*-axis) of all the transcripts (*B*, *left*) and all the proteins (*C*, *left*) in the common set. Horizontal red line indicates the significance cutoff of FDR < 0.01. Vertical black line is at 0 and included for reference. Age effects are reported as log_2_ fold change in abundance per year. The colored points on the volcano plots represent the five most significant (FDR < 0.05) up-regulated enrichment categories and the five most significant down-regulated categories from the gene-set enrichment analysis. Gray points represent transcripts or proteins that were not annotated to the highlighted categories. Top enrichment categories are shown in *B* (*right*) and *C* (*right*), with the enrichment score plotted on the *x*-axis and category on the *y*-axis, point color indicates the adjusted *P*-value, and point size indicates the size of the category. Vertical red line at 0 is included for reference.

Transcripts and proteins are quantified with different technologies and different measurement scales. To understand how age-related changes compare between transcripts and proteins, it is helpful to use sex differences as a point of reference. Histograms of *P*-values for tests of age effects and sex differences (Fig. 2A) show that the smaller number of age-related transcripts compared to proteins is not a result of a difference in statistical power. The shapes of the *P*-value distributions and proportions of significant transcripts are unchanged when we look at the full data (Supplemental Data S3, S4). For transcripts, sex is a stronger driver of differential expression compared to age (887 genes compared to 206 genes at FDR < 0.01). For proteins, age is a stronger driver compared to sex (2084 genes compared to 408 genes at FDR < 0.01). Thus the number of proteins showing significant change with age is greater that the number of transcripts changing with age. The reverse is true for sex differences, which have a greater impact on transcripts. Only three transcripts (*Hspa1b*, *Gm4841*, *Smpx*, FDR < 0.01) display significant sex-by-age interactions, and a larger number of proteins show significant interactions (144 genes, FDR < 0.01).

The most statistically significant age-related transcript is *Ryr1* (Fig. 2B), a calcium release channel found in the sarcoplasmic reticulum of skeletal muscle (Conti et al. 1996). The top 50 age-related transcripts (by absolute value of age effect) include genes related to mitochondrial metabolism (*Ucp1*, *Cyp2b10*), cholesterol transport (*Apoa1*, *Apoa2*, *Apoc1*), immunoglobulin chain components (*Ighg1*, *Igha*, *Ighg2b*, *Igkc*, *Ighg3*, *Ighg2c*, *Igkv1*-*135*), coagulation (*F9*, *F10*, and *F2*), and extracellular matrix remodeling (*Serpina3k*, *Fgg, Fga*, *Serpina1a*, *S100a9*, *Serpina1d*, *Serpina1e*, *Serpina1b*, *Serpina3m*, *Fetub*) (Supplemental Data S1). Box plots of selected transcripts illustrate the magnitude of these changes relative to individual variation across the DO mice (Supplemental Fig. S1).

The most statistically significant age-related protein is SEPSECS (Fig. 2C), Sep (O-phosphoserine) tRNA:Sec (selenocysteine) tRNA synthase, which has been associated with aging and cardiac oxidative stress (Rizvi et al. 2021). Among the top 50 age-related proteins are genes in the mitochondrial respiratory complex I (NDUFA1), complex III (MT-CYTB, UQCRH), complex IV (COX7B), complex V (MT-ATP6), and other mitochondrial functions (OPA3, MRS2, CHCHD4, POLG); proteins involved in immune response (CD81, CD47); extracellular matrix remodeling (FERMT2, COL1A1, COL1A2); immunoglobulin chain components (IGHG2C, IGHA); protein–protein interaction (WDR65, ARVCF); actin cytoskeleton (ACTN3); autophagy and mitophagy (FAM134C, RABGEF1); and regulation of transcription and chromatin remodeling (ZFP947, HOPX, MED23, TRRAP) (Supplemental Data S2). Box plots of selected proteins illustrate these effects (Supplemental Fig. S1).

Age-related changes in bulk tissue analysis can reflect changes in cell composition. Comparing our age-related transcripts with published single-cell RNA-seq data from the heart (Forte et al. 2020), we observe an increase in the myofibroblast marker *Postn* (age effect = 0.58) (Supplemental Data S1). We also observe indications of immune cell infiltration but only for transcripts in the full set for which we do not have protein data. These include markers for B cells (*Cd79a*, age effect = 0.85), macrophages (*Cd68*, age effect = 0.43), and monocytes (*Plac8*, age effect = 1.58) (Supplemental Data S3). Comparing the age-related proteins reported here, we see an increase in markers of smooth muscle (VTN, age effect = 0.58), epicardium (CLU, age effect = 0.38), and endothelium cells (FABP4, age effect = 0.40 and PECAM1, age effect = 0.33). These changes are consistent with the inflammatory and proliferative stages of cardiac healing after injury (Forte et al. 2018).

To further investigate the biological functions of transcripts and proteins that change with age, we ran gene-set enrichment analysis using FGSEA (Korotkevich et al. 2016) based on Gene Ontology (GO) categories (The Gene Ontology Consortium et al. 2000; The Gene Ontology Consortium 2021) for biological process, molecular function, and cellular compartment (Methods). FGSEA is a score-based enrichment approach that does not rely on arbitrarily thresholded gene lists. This proved helpful owing to the substantial difference in the numbers of statistically significant transcripts and proteins. Using the standardized age effect (age effect/SE) as a score, we found 83 significant (FDR < 0.05) enriched categories for transcripts and 26 categories for proteins (Supplemental Data S5, S6). The top five categories for age-related transcripts that increase with age are associated with exocytosis and immune response (Fig. 2B). The top five categories for age-related transcripts that decrease with age are involved in protein modification and folding (Fig. 2B). The top five categories for proteins that increase with age relate to protein transport and autophagy (Fig. 2C). Proteins decreasing in abundance with age are related to fatty acid oxidation, glucose catabolism, muscle structure, and protein folding (Fig. 2C). Enrichment results for the full sets of transcripts and proteins are similar (Supplemental Data S7, S8).

We found enriched categories (FDR < 0.05) for sex and age-by-sex interaction effects. For sex effects, the most significant categories for both transcripts and proteins relate to mRNA metabolism and gene expression regulation. Age-by-sex interaction effects for proteins and transcripts relate to mitochondrial matrix, cellular respiration, mitochondrial gene expression, and mRNA metabolism. Enrichment categories for sex and age-by-sex interaction effects are reported in Supplemental Data S5 and S6.

**Figure 3.**
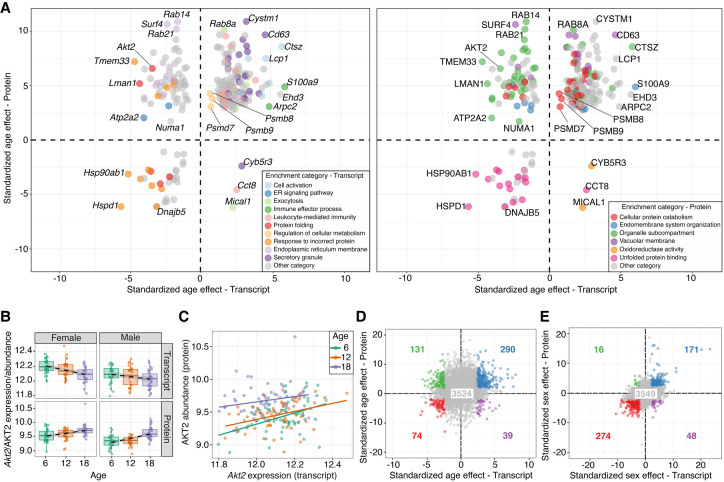
Comparisons of age effects on transcripts and proteins reveal similarities and differences. (*A*) Standardized age effects for transcripts (*x*-axis) and proteins (*y*-axis) with genes highlighted based on enrichment analysis results from the transcriptome (*left*) and proteome (*right*). Colored points represent enrichment categories of genes based on enrichment sets defined by transcripts (*left*) and by proteins (*right*). Gray points represent genes that were not annotated to one of the highlighted sets. Horizontal and vertical lines at 0 included for reference. (*B*) Change in transcript (*top*) and protein (*bottom*) abundance (*y*-axis) of the gene *Akt2* with age (*x*-axis), stratified by sex (with females on *left* and males on *right*). Best fit lines included to emphasize trends. (*C*) Protein abundance by transcript expression for the gene *Akt2*. Points are colored based on age group. Best fit lines for each age group included to illustrate correlation. (*D*) Standardized age effects for all the proteins (*y*-axis) plotted against the effect for their corresponding transcripts (*x*-axis). Blue points represent genes that have concordant increases with age for both transcripts and proteins. Green points represent genes that have discordant decreases with age for transcripts but increases for proteins. Purple points represent genes that have discordant increases with age for transcripts but decreases for proteins. Red points represent genes that have concordant decreases with age for both transcripts and proteins. Gray points represent genes that do not have a significant age effect (FDR > 0.1) for both transcript and protein. The number in each quadrant corresponds to the number of total genes in each group. Horizontal and vertical lines at 0 included for reference. (*E*) Standardized sex effects for all the proteins (*y*-axis) plotted against the effect for their corresponding transcripts (*x*-axis). Points colored as in *D* with the corresponding number of total genes in each group.

Comparison of functional enrichment categories for the age-related transcripts and proteins identifies only one category in common (GOMF_UNFOLDED_PROTEIN_BINDING). However, by merging the enrichment tables based on gene IDs instead of GO categories, we find 245 genes in common that are annotated to different (but related) categories in each set of enrichment results (Fig. 3A; Supplemental Data S9). For example, some of the genes annotated as *immune response* and *exocytosis* for proteins are annotated as *vacuolar membrane* and *organelle subcompartment* for transcripts (Fig. 3A). These genes include *S100a9*, *Cd63* and the genes encoding subunits of the immunoproteasome (*Psmb8* and *Psmb9)*, all of which increase with age for both transcripts and proteins (Fig. 3A; Supplemental Data S1, S2). Transcripts annotated to response to *incorrect protein* correspond to proteins that are annotated to *unfolded protein binding* (Fig. 3A). These genes include heat-shock proteins coding, such as *Hsp90ab1*, *Hspd1*, and *Dnajb5*, and they all decrease with age for both transcripts and proteins (Fig. 3A; Supplemental Data S1, S2). Thus, transcripts and proteins provide consistent indicators for increase in immune response and for decrease in protein folding with age.

A number of transcripts and proteins that share functional annotations change in opposite directions with age. The genes *Cyb5r3*, *Cct8*, and *Mical1* are annotated in the *secretory granule*, *leukocyte-mediated immunity*, and *exocytosis* categories for transcripts, but for proteins, they fall into the *oxidoreductase activity* (*Cyb5r3* and *Mical1*) and *unfolded protein binding* (*Cct8*) categories (Fig. 3A). All three genes show increase transcript abundance but decrease protein abundance with age (Fig. 3A; Supplemental Data S1, S2). Some of the genes annotated in categories related to *endoplasmic reticulum membrane* and *response to incorrect protein* for transcripts are annotated as *organelle subcompartment* and *vacuolar membrane* for proteins (Fig. 3A). These include the genes *Rab14*, *Rab21*, and *Akt2* that decrease for the transcript data but increase for the proteins with age (Fig. 3A; Supplemental Data S1, S2), suggesting that the age-related up-regulation of genes involved in intracellular protein transport and vesicle formation is seen only for proteins.

Transcripts and proteins that change in opposite directions with age are often positively correlated within age groups. For example, the transcript of *Akt2* decreases with age, whereas the protein AKT2 increases with age (Fig. 3B), and yet they are positively correlated across all age groups (6 mo, r = 0.411, *P* = 0.00091; 12 mo, r = 0.375, *P* = 0.0027; 18 mo, r = 0.216, *P* = 0.095) (Fig. 3C). We observed many examples of genes for which the transcript and protein abundances change in opposite directions despite significant positive correlation within age groups, such as *Cyb5r3* (Supplemental Fig. S2). A global comparison of the standardized age effects for transcripts and proteins reveals that the age effects are positively correlated (r = 0.128, *P* < 2.2 × 10^−16^) (Fig. 3D) but less so in comparison to the correlation of sex differences (r = 0.379, *P* < 2.2 × 10^−16^) (Fig. 3E). We observe that the within-gene variances of transcripts increase continuously with age, and that the within-gene variances of proteins increase but with an inflection between 12 and 18 mo of age (Supplemental Fig. S3A,B). For the majority of genes, there is positive correlation between the transcript and protein abundances within age groups and a reduction in average correlation for the 18-mo age group (6 mo, median r = 0.175; 12 mo, median r = 0.171; 18 mo, median r = 0.148) (Supplemental Fig. S3C). Thus, we see an increase in variability and a corresponding reduction in correlation between transcript and proteins with age, but the reduction in correlation is small. For most genes, transcript and protein are positively correlated across all ages. This persistence of positive correlation suggests that age-related changes in post-transcriptional regulation of proteins can shift the balance between transcript and protein abundances as animals age and can lead to discordant (or concordant) age effects between a transcript and its protein product without uncoupling of the positive correlation between them.

### Loss of stoichiometry occurs across multiple protein complexes in the aging heart

Loss of stoichiometry in protein complexes has been shown to occur with age in a number of tissues and organisms (Ori et al. 2015; Anisimova et al. 2018; Kelmer Sacramento et al. 2020; Taggart et al. 2020). We examined protein complexes defined in Ori et al. (2016) (Methods). We considered only complexes for which we have both transcript and protein data for four or more subunits, a total of 123 out of the 279 annotated complexes. We computed Pearson's correlations between all pairs of proteins and between all pairs of transcripts within each protein complex. To assess overall change with age for an entire complex, we fit a joint regression model to the within-complex correlations with a random intercept term for each gene pair and a common slope to capture the average change in correlation per year across all pairs of subunits in the complex (Methods). We evaluated the significance of these effects using a permutation procedure (McKenzie et al. 2016).

**Figure 4.**
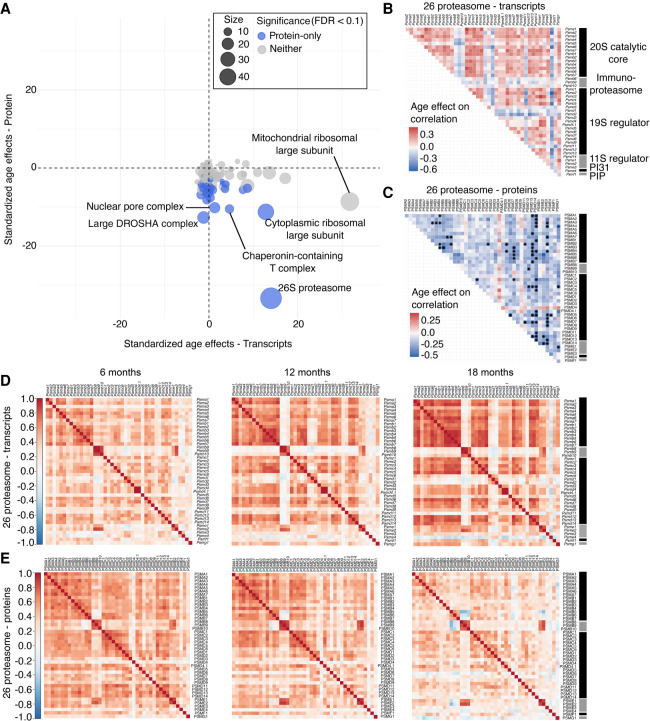
Correlations between protein complex members change with age. (*A*) Standardized age effects on correlation for 123 protein complexes are shown for transcripts (*x*-axis) and proteins (*y*-axis). Point size represents the number of proteins observed for each complex. Blue points represent protein complexes with a significant age effect (FDR < 0.1) for proteins. Gray points represent protein complexes without a significant age effect for transcript or protein. Age effects are estimated by linear regression and reported as change in the correlation coefficient per year. Horizontal and vertical lines at 0 included for reference. Heatmaps represent the age-related change in correlations between gene pair members of the 26S proteasome complex for protein abundance (*B*) and transcript expression (*C*). Dots indicate significant pairwise changes in correlations with age (FDR < 0.1). Black and gray bars on the *y*-axis indicate subcomplexes of the 26S proteasome. Heatmaps represent the Pearson's correlation matrices for 26S proteasome complex for transcripts (*D*) and for proteins (*E*) at 6 mo, 12 mo, and 18 mo (*left* to *right*).

Although we did not observe any significant changes in overall correlation among transcripts, we identified multiple complexes that change in overall correlation among proteins. We found that for 107 out of the 123 protein complexes, the average correlation between protein pairs decreases with age (Fig. 4A; Supplemental Data S10). Of these, 40 reached statistical significance (FDR < 0.1). The complexes with the largest standardized age effects (shown here as change in average correlation per year ± SE) include the nuclear pore complex (NPC) (protein age effect = −0.25 ± 0.02), chaperonin-containing T complex (TRiC) (protein age effect = −0.28 ± 0.03), cytoplasmic ribosomal large subunit (protein age effect = −0.13 ± 0.01), large DROSHA complex (protein age effect = −0.29 ± 0.02), and the 26S proteasome complex (protein age effect = −0.20 ± 0.006) (Fig. 4A). There is no obvious relationship between change in correlation and change in protein abundance with age. For the proteasome complex, we see that most of the subunits increase in abundance with age (Supplemental Fig. S4). For example, proteins PSMB3 and PSMD7 both increase in abundance with age but decrease in their correlation with age (Supplemental Fig. S4). We examined age-specific correlation within complexes separately for each sex. There appears to be a tendency for transcripts to increase in correlation with age more for females, but none of the overall changes are statistically significant (Supplemental Fig. S5). For proteins, we observed consistent decreases in correlation for both females and males across most of the complexes (Supplemental Fig. S6).

To determine if the change in correlation of proteins with age is specific to the heart we repeated this analysis on data from kidney of the same DO mice (Takemon et al. 2021). In kidney, there is a modest trend of increased correlation with age for proteins (Supplemental Fig. S7A), suggesting that these age-related declines in protein complex correlations are tissue specific. The protein complexes with the largest age effects in the kidney are the NURD complex (protein age effect = 0.40 ± 0.04), emerin C32 (protein age effect = 0.19 ± 0.02), spliceosome A (protein age effect = 0.19 ± 0.02), spliceosome U2 (protein age effect = 0.24 ± 0.02), large DROSHA complex (protein age effect = 0.19 ± 0.03), and mitochondrial pyruvate dehydrogenase complex (protein age effect = 0.31 ± 0.05) (Supplemental Fig. S7A; Supplemental Data S10). These differences between heart and kidney can be explained by looking at the correlation between transcript/protein age effects in the kidney and transcript/protein age effects in the heart. Transcript changes with age in the heart and kidney are highly correlated (r = 0.45); however, this is not true for proteins (r = 0.027) (Supplemental Fig. S7B). This observation suggests that the post-transcriptional regulation of protein complex stoichiometry is tissue specific, but it could also reflect different rates of change with age across tissues.

To take a closer look at how correlation changes between specific protein pairs within a complex, independent of complex size, we applied the same permutation test to evaluate individual gene pairs (Methods).

Among transcripts, 60 pairs (out of 4396) had significant age-related changes in correlation (FDR < 0.1), representing 22 different protein complexes (Supplemental Data S11). The complexes with the highest proportion of significant transcript pairs with significant changes are the ubiquilin-proteasome complex (60%), the TNF/NFKB1 signaling complex (33%), the SMG-1-Upf1-eRF1-eRF3 (SURF) complex (33%), the mitochondrial complex V (23%), and the COP9 signalosome complex (18%) (Supplemental Fig. S8). The majority (53 out of 60) of significant transcript pairs with significant age-related changes to correlation increases with age.

We identified 351 protein pairs (out of 4396) with significant age-related changes in correlation with age (FDR < 0.1) (Supplemental Data S11). These pairs span 50 protein complexes, and the complexes with the largest proportion of significant pairs (significant number pairs/total number of pairs) are the TRiC (69%), Suv39h1 histone complex (67%), dynactin complex (40%), multi-eIF complex (39%), and the large DROSHA complex (39%) (Supplemental Fig. S8). Although not among the top protein complexes in terms of proportion of significant pairs, we found that many protein pairs changing within the 26S proteasome decrease in correlation with age (Fig. 4B,C), consistent with our evaluation of overall changes within the proteasome complex (above). The protein PSMD14 was the most common (part of 16 pairs), followed by proteins PSMD13 and PSME3 (11 pairs) (Fig. 4C). Proteins from the PSMC and PSMD families constitute the 19S particle of the proteasome, but the PSME family is part of the 11S particle. Both of these particles are regulators of the 26S proteasome complex, suggesting that the age-related changes in correlation is affecting the regulatory subunits of the proteasome. The correlation patterns for the inducible immunoproteasome subunits (PSMB8, PSMB9, and PSMB10) are consistent across age groups for both transcripts and proteins (Fig. 4D,E). Overall, the majority (319 out of 351) of the significant protein pairs showed reduced correlation with age, consistent with a global loss of stoichiometric balance in these protein complexes.

### Genetic variants alter the age trajectory of proteins that are associated with proteostasis

We performed genetic mapping for the full sets of transcripts and proteins to identify quantitative trait loci (eQTL and pQTL, respectively). The additive effects of genetic variation on transcripts and proteins have been widely documented (Hause et al. 2014; Battle et al. 2015; Chick et al. 2016; Albert et al. 2018; Yao et al. 2018) and are not discussed here, but we have made the mapping results available for others to explore. Here, we focus on how genetic variants influence the rate or direction of change with age of transcripts and proteins. To identify these age-interactive QTL (age-QTL), we tested for an age-by-genotype interaction term in a linear mixed model for each transcript and protein (Methods). We computed a significance threshold for age-interaction LOD score that controls the genome-wide false positive rate at 0.05 (LOD_int_ > 7.75). The data, along with the additive and age-interactive QTL results, can be explored interactively or downloaded at our QTL Viewer website (https://qtlviewer.jax.org/agingheart). A user's guide for the QTL Viewer is available at https://qtlviewer.jax.org/userguide.

We identified 1035 transcript age-QTL (age-eQTL) (Supplemental Data S12). Most age-eQTL mapped to locations that are distant from their coding genes, suggesting that the effect of genetics on age-related changes is not mediated directly through the coding gene but rather occurs in response to other factors that change with age. There are, however, six local age-eQTL for the genes *Rin3*, *Spryd7*, *Rhbdl3*, *Gm9925*, *Sstr3*, and *Ttyh1*. The local age-eQTL with the highest LOD score is for the gene *Rin3* (LOD_int_ = 9.06), located on Chromosome 12. *Rin3* is a guanidine nucleotide exchange factor and functions as a stabilizer for proteins of the RAB5 family, which regulates endocytosis and intracellular vesicular trafficking (Bucci et al. 1992; Kajiho et al. 2011).

We identified 603 protein age-QTL (age-pQTL) (Supplemental Data S12) and, similar to the age-eQTLs, most of the age-pQTL are distal. The only local age-pQTL is for FN3KRP, located on Chromosome 11, a fructosamine 3 kinase involved in the reversal of the nonenzymatic glycation of proteins (Collard et al. 2003). Glycation is a process of protein oxidation that makes proteins less functional and active (Szwergold et al. 2011). We note that *Fn3krp* was recently associated with longevity in humans (Torres et al. 2021).

**Figure 5.**
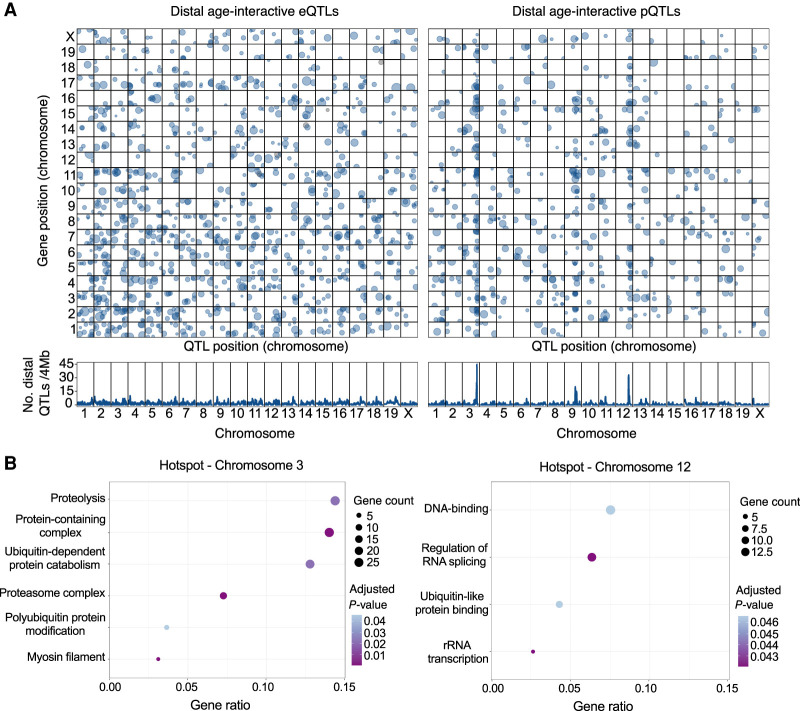
Genetic mapping reveals genomic hotspots of age-interactive QTL. (*A*) Age-interactive QTL (age-QTL) were identified by testing for an age-by-genotype interaction effect and are plotted for transcripts (age-eQTL, *left*) and protein (age-pQTL, *right*). Significant age-QTL (genome-wide error rate < 0.05) are plotted (*top*) based on the location of their peak association (*x*-axis) and the position of the coding gene (*y*-axis). Point size is proportional to LOD score. The *lower* panels show the density of distal age-QTL detected at positions spanning the genome based on 4-Mb windows. Two hotspots for distal age-pQTLs were identified on Chromosomes 3 and 12. (*B*) Functional enrichment results for proteins that map age-pQTL to the hotspots on Chromosome 3 (*left*) and Chromosome 12 (*right*). Enrichment categories are plotted by with their gene ratio—the number of genes in hotspot-defined sets in category divided by the number of all the genes in the category—with point size indicating the number of genes and point color indicating the adjusted *P*-value.

Many of the significant age-pQTL colocate to the genome in hotspots on Chromosomes 3 (270 proteins) and 12 (224 proteins) (Fig. 5A). To consider weaker associations but potentially biologically relevant proteins, we expanded the proteins in each hotspot to include suggestive age-pQTL (LOD_int_ > 6). We then filtered the candidate proteins by retaining only those with absolute mean correlation greater than 0.3 with other members of the hotspot. This filter removed genes with age-pQTL that are not tightly correlated with other genes at the hotspot and thus less likely to share a common genetic regulator. After filtering, the age-pQTL hotspot on Chromosome 3 includes 208 proteins, and the Chromosome 12 hotspot includes 194 proteins (Supplemental Fig. S9; Supplemental Data S12).

To determine if proteins that map to the age-pQTL hotspots share common biological functions, we performed enrichment analysis using clusterProfiler (Yu et al. 2012). The Chromosome 3 hotspot has a total of 12 enriched gene sets (FDR < 0.05) (Supplemental Data S13). From these, 10 relate to protein modification processes, including ubiquitination and proteolysis (Fig. 5B; Supplemental Data S13), including proteins from the proteasome 20S catalytic core (PSMA3 and PSMB6) and the regulatory particle (such as PSDM11 and PSMD14). The Chromosome 12 hotspot, has six enriched categories (FDR < 0.05) with functions related to transcription regulation and chromatin organization, including histone H2 family members (such as MACROH2A1 and MACROH2A2) and histone chaperones (NPM1) (Fig. 5B; Supplemental Data S13). It also includes proteins related to proteasomal degradation, such as DNAJB2 and RAD23B. We note that RAD23B and 32 other proteins are found in both hotspots, including proteins associated with muscle cell structure (MYH3, MYH6, AND MYH7B), vesicle formation and transport (SNAP29, DCTN2, CLTA, and CHMP4B), and proteins related to protein modification and degradation pathways (PSMD4, NSFL1C, and UBXN1). Although these age-pQTL loci should be considered as tentative until they can be independently validated, the predominance of proteins that are constituents of heart muscle and/or play a role in protein quality control suggest that these functions are genetically malleable and could contribute to individual variability in heart aging.

## Discussion

In this study, we examine the changes that occur with aging in the transcriptome and proteome of heart tissue from 185 genetically diverse mice. Most mice in this study are still healthy at 18 mo of age, and thus the age-related changes reflect normal aging, free of major pathologies. We analyze functional annotations of age-related transcripts and proteins and identify biological processes that are altered through the course of natural aging. We identify changes in transcript and protein abundance in bulk heart tissue, however, we are unable to identify compartmentalized changes that could be achieved with single-cell studies.

A dominant feature of transcriptal change is an increase in the expression of genes associated with immune response—some of these changes are also seen in proteins. Increase in immune cell composition across multiple tissues (Müller-Werdan 2007; Moro-García et al. 2013) and up-regulation of immune response genes in the aging murine heart are well documented (Bartling et al. 2019; Greenig et al. 2020). We observe a marked increase in complement factor genes that is also seen in our previous study of aging kidney on the same mice (Takemon et al. 2021) and in the proteome of human plasma (Johnson et al. 2020). Complement component 4B (Chido blood group) (*C4b*) is one of the transcripts with the highest change with age in our data. In addition, we see an increase in marker genes for leukocytes, which play an important role in the adaptive immune response. In the aging heart, leukocytes mediate early stage physiological changes (Ramosa et al. 2017), and the increased leukocyte signature seen here in healthy aging mice corroborates their role early in the aging process.

Genes associated with vesicle membranes and exocytosis, responsible for secretion and cross-talk between cells, are up-regulated with age (mostly for proteins). Some of these changes reflect immune cell signaling, but we also see changes in extracellular matrix remodeling that are typical in age-related hypertrophy (Chiao and Rabinovitch 2015). These genes include *Ahsg*, *Serpinf2*, and *Serpina3n* (Kang et al. 2017; Santos-Lozano et al. 2020). The gene *Serpinf2* plays a role in fibrinolysis protecting against blood clotting, and SERPINF2 protein is found in higher levels in the plasma of healthy centenarians and is associated with healthy aging (Santos-Lozano et al. 2020). The serine/threonine kinases AKT1 and AKT2 are also involved in cellular transport processes (Greenig et al. 2020) and both increase with age. These proteins are highly abundant in cardiomyocytes where they are involved in the regulation of cardiac hypertrophy in aging through interaction with sirtuins (Pillai et al. 2014). We see changes across proteins from the RAB family, Ras-like GTPases that regulate protein trafficking by vesicle formation and fusion throughout the cell (Martinez and Goud 1998; Wu et al. 2001). Among the 39 RAB proteins that significantly change with age, 32 increase (Supplemental Data S2), indicating the activation of cellular secretion and transport pathways with age, which is commonly observed in hypertrophic hearts (Chiao and Rabinovitch 2015).

Transcripts and proteins related to protein folding decrease with age (Figs. 2A,B, 3A). These include heat-shock proteins (HSPs), molecular chaperones that inhibit unfolding or denaturation of proteins in response to stress. HSPs act to limit the accumulation of damaged proteins and subsequent proteotoxicity in aging cells (Murshid et al. 2013). Some proteins, including HSPA5, HSP90B1, and PDIA4 decrease with age with no corresponding change in their transcripts. These are downstream targets of the ATF6 branch of the endoplasmic reticulum stress response (Higuchi-Sanabria et al. 2018). The activation of this pathway in the heart increases responses that correct misfolded proteins and avoid apoptosis (Toko et al. 2010; Higuchi-Sanabria et al. 2018). Their age-related decline suggests the disruption of early steps within the protein quality control system that can lead to a pathological instead of an adaptive response to unfolded proteins (Szegezdi et al. 2006; Toko et al. 2010).

Enrichment analysis of proteins that change with age highlights additional biological functions that are not seen for the transcriptome. Proteins associated with fatty acid oxidation and glucose catabolism decrease with age (Fig. 2C). The metabolic shift from fatty acid to glucose is known to occur in the aging heart (Strait and Lakatta 2012), however, here, we show a decline in glycolysis-related proteins as well. We note that decreased glycolysis has been reported in hypertrophied hearts (Tran and Wang 2019). In addition, the proteome data show activation of autophagy pathways with age (Fig. 2C). This may be compensatory for the decrease in protein folding pathways. Autophagy increases when the cellular protein quality control system is overwhelmed (Nakai et al. 2007) and it is crucial for maintaining cardiac function (Taneike et al. 2010).

We confirm previous studies showing that age-related transcriptional changes are not necessarily mirrored in their protein products, and likewise, age-related changes in proteins are not necessarily preceded by transcriptional changes (Waldera-Lupa et al. 2014; Wei et al. 2015; Kelmer Sacramento et al. 2020; Takemon et al. 2021). In many cases, transcripts and their corresponding proteins change in opposite directions with age (Fig. 3D). These discordant age-related changes occur even when there is persistent positive correlation of transcript and protein abundances within age groups. This points to a shift in the balance between transcript and protein because of changes in the post-transcriptional mechanisms that regulate protein abundance. This is supported by our observation that functional categories of age-related change, especially for proteins, are enriched across post-transcriptional and post-translational protein regulatory processes.

In addition to age-related changes in protein abundance, we observe a widespread decrease with age in the correlation between proteins within complexes, suggesting loss of stoichiometry. These changes are most pronounced for complexes involved in different steps of protein homeostasis, including the TRiC, cytoplasmic ribosomal large subunit, NPC, large DROSHA complex, and the 26S proteasome. After transcription, messenger-RNAs are transported into the cytoplasm through the NCP complex (Strambio-De-Castillia et al. 2010). The NPC is an aqueous channel that regulates nuclear permeability and deterioration with age leads to leakage of cytoplasmic proteins into the nucleus (D'Angelo et al. 2009). Transcripts in the cytoplasm can be modified post-transcriptionally by micro-RNAs (miRNAs), which are primarily processed by the DROSHA complex, a RNase that cleaves primary miRNAs releasing hairpin-shaped ones (Han et al. 2004). Following translation of mRNA by the ribosome complex, proteins are folded into three-dimensional structures assisted by the TRiC. Although TRiC is responsible for folding only 5%–10% of the mammalian proteome, it plays a special role in muscles where it regulates sarcomere assembly (Berger et al. 2018). Defects in TRiC proteins have been associated with skeletal muscle defects and myocardial infarction (Berger et al. 2018). Misfolded proteins that cannot be fixed by TRiC are degraded by the 26S proteasome, autophagy, and lysosomes. Our findings highlight the impacts of aging on multiple complexes involved in protein homeostasis (synthesis, transport, folding, and degradation) in the murine heart.

The 26S proteasome complex showed the most significant decline in correlation with age (Fig. 4A). This complex has 43 protein subunits, which gives us enough power to detect subtle changes. However, other large protein complexes (e.g., the mitochondrial ribosomal large subunit) did not show the same level of correlation loss. The 26S proteasome plays essential roles in the heart, including the turnover of gap-junction and contractile proteins (Li and Wang 2011), and NFKB1 and CTNNB1 signal transduction in cardiac remodeling (Palombella et al. 1994). The 26S proteasome subunits related to the 19S and 11S regulatory particles (PSMD and PSME families) show the steepest decline in correlation among individual gene pairs (Fig. 4C). These “gates” of the proteasome regulate binding to either ubiquitinated (19S) or non-ubiquitinated (11S) proteins and control entrance to the degradation cascade (Coux et al. 1996). It is described that proteasome activity declines with age in many tissues (Carrard et al. 2002; Predmore et al. 2010). One hypothesis is that reduced expression of proteasome subunits leads to the decline of proteasome activity, however, disruption in protein degradation can occur without reduction in proteasome abundance (Predmore et al. 2010). We did not observe a reduction in the abundance of subunits from the proteasome complex in heart, in fact, most of them increase with age (Supplemental Fig. S4A). An alternative hypothesis is that post-translational modifications and an excess of oxidized proteins contribute to the decline in proteasome activity in the heart (Predmore et al. 2010). Here, supported by previous findings (Ori et al. 2015; Kelmer Sacramento et al. 2020), we propose that age disrupts the balance of proteasome subunits in the heart, which contributes to a cycle of progressive breakdown of protein homeostasis during the aging process. In contrast to the rest of the 26S proteasome, the immunoproteasome-specific subunits PSMB8, PSMB9, and PSMB10, maintain positive correlation across all ages for both transcripts and proteins (Fig. 4D,E). On the other hand, the immonoproteasome becomes more anti-correlated with their constitutive partners (PSMB5-7) with age. The anti-correlation between immunoproteasome and the constitutive subunits was also observed in the liver tissue from DO, Collaborative Cross, and founder mice, and it was shown to be partially modulated by genetic factors (Keele et al. 2021). The immunoproteasome supports the generation of peptides for antigen presentation by MHC class I molecules (Basler et al. 2013) and plays a role in the activation of adaptive immune pathways (Basler et al. 2013). The age-related maintenance of the correlation within the immunoproteasome and the increase in the anti-correlation between their subunits and their constitutive parners may reflect the increase in immune cells in the heart of older mice.

The production of protein complex subunits is not always perfectly stoichiometric, and post-translational mechanisms play an essential role in removing extra subunits, promoting balance of the components of complexes (Taggart et al. 2020). The age-related loss of stoichiometry that we show here are mediated by post-transcriptional mechanisms supported by the very same protein complexes that are affected. This suggests a feedback loop that could accelerate decline. We did not observe loss of stoichiometry in these complexes in kidney tissue from the same mice (Supplemental Fig. S7A). The heart, unlike any other tissue in the body, is under constant mechanical load, which challenges the large structural proteins to maintain their proper conformation and may require more replenishment of proteins (Li and Wang 2011). For this reason, the heart may be more susceptible to proteotoxic stress when the protein quality control system is functioning less than optimally. Age-related molecular changes have different onsets across tissues (Schaum et al. 2020), and these changes may occur later in kidney. Others have also described tissue-specific loss of stoichiometry of protein complexes with age (Ori et al. 2015; Kelmer Sacramento et al. 2020).

A unique advantage of molecular aging studies using genetically diverse mice is the opportunity to map loci that modify the age trajectory of transcripts and proteins. The majority of age-interactive quantitative trait loci (age-QTL) that we identified are distant from the coding genes of the affected transcripts and proteins. Thus, the underlying genetic variants are not acting on the proximal genes but instead exert influence on changes in the cellular environment that occur with aging. We identified two dense clusters of age-pQTL on Chromosomes 3 and 12. The proteins that map to the Chromosome 3 hotspot are enriched for functions in the proteasome complex and protein ubiquitination (Fig. 5B), whereas proteins that map to the Chromosome 12 hotspot are associated with chromatin structure, transcriptional regulation, and ribosome biosynthesis (Fig. 5B). Although the age-QTL reported here should be considered tentative until they can be replicated in other studies, the prevalence of age-pQTL for proteins involved in protein quality control suggests that the post-transcriptional mechanisms regulating protein homeostasis during aging are modulated by genetic variants that could contribute to individual variation in the rate of cardiac aging.

In summary, we observe changes associated with healthy aging in the murine heart that suggest a scenario of activation of immune response, hypertrophy, and dysregulation of protein homeostasis. We show that the transcriptome alone is not able to reveal the full spectrum of changes in the aging heart, which underscores the importance of post-transcriptional regulation of proteins in aging. The proteome data uniquely reveal features including metabolism changes and the activation of autophagy pathways with age. We observe a loss of stoichiometry among protein complexes, especially those that are involved in protein homeostasis. We see many indications that the protein quality control system becomes disrupted with age, starting from the reduced expression of proteins involved in handling misfolded proteins to the loss of stoichiometry of the proteasome complex. We find evidence that genetic variation can influence the age-related dynamics of protein homeostasis. Our findings illustrate how transcriptome and proteome profiling of genetically diverse mice can reveal broad patterns of change in the molecular dynamics of the aging heart. The data generated by this study and our previous study of the aging kidney (Takemon et al. 2021) provide resources that can be mined to obtain additional insights into tissue-specific molecular processes associated with aging.

## Methods

### Study cohort and tissue collection

A cross-sectional aging study was performed with 600 DO mice (300 of each sex) bred at the Jackson Laboratory (stock no. 009376) across breeding generations 8–12 of the DO stock. Mice were maintained on a standard rodent chow (LabDiet 5K52) in an animal room that was free of pathogens, had a set temperature ranging from 20°C to 22°C, and a 12-h light/dark cycle. Animals were housed four to a pen and pens were randomly assigned to 6-, 12-, and 18-mo age groups. Whole hearts were dissected, flash-frozen, pulverized, and aliquoted. A subset of 192 samples, balanced across age groups and sexes, were selected for RNA-seq and shotgun mass spectrometry. The mass spectrometry was performed for 190 of the 192 mice. The Jackson Laboratory Institutional Animal Care and Use Committee approved all procedures used in the study. The mice were selected by requiring that all had genotype, transcript, and protein data, leaving a total of 185 animals for analysis.

### Power to detect age effects and QTL in study

The sample size required to detect a significant age effect is determined by the size of the effect (difference in means between the 6- and 18-mo age groups) relative to the variance within age groups. Therefore, we define the strength of an effect in units of SD of the within group variance. Based on standard power calculations (Wilson Van Voorhis and Morgan 2007), with a sample size of about 64 animals per age group we can expect 0.80 power to detect an age effect of 0.5SD at an unadjusted type I error of 0.05. In practice, because of the sample size and the high precision of the RNA and protein quantification, we were able to detect age effects in a large proportion of genes even after applying a false discovery rate correction for multiple testing (Holm 1979). To evaluate the power for genetic mapping, we referred to simulations conducted by Gatti et al. (2014). After applying genome-wide error rate correction to account for the multiple testing burden incurred from genome-wide scans, our sample of 185 animals has expected power of 0.80 to detect a QTL that explain 0.20 of the total variation in RNA or protein expression.

### Bulk RNA extraction

Frozen and pulverized heart tissue was lysed in Ambion TRIzol reagent (Thermo Fisher Scientific 15596026). Bulk RNA was isolated using the miRNeasy Mini kit (Qiagen 217004), according to the manufacturer's protocols with the DNase digest step. RNA concentration and quality ratios were assessed using the NanoDrop 2000 spectrophotometer (Thermo Fisher Scientific) and RNA 600 Nano LabChip assay (Aligent Technologies).

### RNA sequencing and quantification

Poly(A) RNA-seq libraries were generated using the TruSeq Stranded mRNA Library Prep Kit (Illumina). Libraries were pooled and 100-bp single-end reads were sequenced on the HiSeq 2500 (Illumina) using TruSeq SBS Kit v4 reagents (Illumina). The RNA-seq experiment was performed in two replicates for each sample distributed across eight lanes. The replicates of each sample were performed in different lanes to avoid lane effects.

We used the Genotype by RNA-seq (GBRS) software package (https://gbrs.readthedocs.io/en/latest/) to generate both total gene counts and genotype probabilities. The process involved combining single-end FASTQ files per sample and aligning to a hybridized (eight-way) transcriptome generated for the eight DO founder strains. The mouse genotype probabilities were reconstructed along approximately 69,000 GigaMUGA markers and used to confirm genotypes and identify mislabeled samples in the quality control process (described in detail below). Gene expression quantification used the Expectation-Maximization algorithm for Allele Specific Expression (EMASE) (Raghupathy et al. 2018), which was then used to quantify allele-specific and total gene counts from RNA-seq data. Transcripts were removed from further analysis if they did not have at least one read in at least half of the samples, resulting in a total of 21,016 transcripts being analyzed. Total gene counts were normalized relative to total read counts using the variance stabilizing transform (VST) as implemented in DESeq2 (Love et al. 2014). For the genetic mapping analysis only, to minimize the impact of outliers, we transformed the VST normalized data to rank normal scores (Conover and Iman 1981).

### Identification of sample mixups

We identified samples that were likely mislabeled by comparing the genotypes (in the form of founder haplotype reconstructions) from GigaMUGA (https://dodb.jax.org/) to reconstructions derived from the gene expression data using GBRS. Founder haplotypes were reconstructed from GigaMUGA using a hidden Markov model implemented in the R/qtl2 package (Broman et al. 2019). Samples could only be compared that had data from both GigaMUGA and RNA-seq, resulting in 189 samples. We first interpolated haplotype reconstructions to the same genomic coordinates. The haplotype reconstructions represent additive founder allele probabilities at positions spanning the genome. For each individual mouse, we averaged across Euclidian distances between the array-based and expression-based reconstructions at loci spanning the genome:

$${\bar{d}}_{i}=\frac{1}{M}\underset{m}{\sum }{\left(\overset{8}{\underset{a\;=\;1}{\sum }}{\left(p_{m,i,a}\;-\;q_{m,i,a}\right)}^{2}\right)}^{1/2}\forall \;i,$$

where *p*_*m*, *i*,*a*_ is the probability estimated from GigaMUGA for marker *m* = 1, …, *M* of founder allele *a* for mouse *i*, and *q*_*m*,*i*,*a*_ is the corresponding probability estimated from gene expression. We flagged animals with ${\bar{d}}_{i}\;>0.8$ as having haplotype reconstructions that differed greatly between the two sources of genetic data and thus potentially represent mislabeling in one of the sources. We identified two mice that upon closer examination likely had swapped IDs in the gene expression data, which we excluded, resulting in 187 mice. Before processing the data for downstream analysis, we also filtered samples to the common set between the transcriptome and proteome data, resulting in a total of 185 mice. This final set included 33 females and 29 males at age 6 mo, 31 females and 31 males at age 12 mo, and 27 females and 34 males at age 18 mo. DO generation 8 was represented by 42 mice, generation 9 by 33 mice, generation 10 by 36 mice, generation 11 by 38 mice, and generation 12 by 36 mice.

### Mass spectrometry and protein quantification

The mass spectrometry (MS) procedure for protein quantification was performed as described in Chick et al. (2016). Briefly, tissue from total heart samples were homogenized in 1 mL lysis buffer, which consisted of 1% SDS, 50 mM Tris, pH 8.8, and Roche complete protease inhibitor cocktail (Roche 11697498001). Peptides were measured using Tandem Mass Tags (TMT), which allows multiple samples to be quantified in a single MS run. Anaysis of all samples was performed in 19 batches with 10 individuals each. Samples were assigned to batches in randomized order to avoid confounding the batch with factors like age and sex. Sample labels were not masked, and no technical replicates were included. MS spectra assignments were made using the Sequest algorithm (Eng et al. 1994) with the Ensembl database (mouse: Mus_musculus NCBIM37.61).

Before protein abundance estimation from their component peptides, we filtered out peptides that contained polymorphic sites across the eight founder strains of the DO. Polymorphisms can induce false pQTL signals because the MS quantification is relative to the B6 mouse reference, resulting in non-B6 alleles being undetected and artificially set to 0 (Zhang et al. 2021). To maximize usage of the data, we first refined the set of polymorphic peptides to be filtered out by confirming that data from polymorphic peptides from the heart clearly matched our expectations based on the founder strain genomes. We used our previous approach of correlating allele effects at putative local QTL for each peptide in the polymorphic set with the B6 allele's distribution pattern among the eight founder strains (Keele et al. 2021). Peptides with a correlation ≥ 0.7, thus matching the expected polymorphism signal, were filtered from the data. For this refinement step, we used heart peptide data from the related inbred Collaborative Cross population, which possesses the same genetic variation as the DO. To estimate and normalize protein abundance from component peptides, we followed Huttlin et al. (2010) and calculated:

(1) $$\mathrm{Protei}n_{ij}=\mathrm{lo}g_{2}\left(\frac{\sum_{K}\mathrm{Peptid}e_{ik}}{s_{i}}+1\right)$$

where *K* represents the set of observed peptides that map to protein *j* for mouse *i*, and *s*_*i*_ is a scaling factor used to standardize samples within a batch.

$$s_{i}=\frac{\sum_{L}\mathrm{Peptid}e_{il}}{\max\left(\sum_{L}\mathrm{Peptid}e_{ql}:Q\in b[i]\right)},$$

where *L* is the set of all peptides observed for a sample, *b*[*i*] denotes the batch of sample *i*, and $\max\left(\underset{L}{\sum }\mathrm{Peptid}e_{ql}\;:\;Q\in b[i]\right)$ is the maximum sum of peptide intensities for all the samples in batch *b*[*i*]. Protein abundance levels that were missing (NA) for more than half the samples were excluded, resulting in a total of 4221 proteins for further analysis.

As we did previously (Keele et al. 2021), batch effects were removed using a linear mixed effect model (LMM) fit with the lme4 package (Bates et al. 2015). The batch effect, estimated as a best linear unbiased predictor (BLUP), was subtracted from each protein abundance, and age (as a categorical variable with three levels) and sex were included as fixed effect covariates in the model. For genetic mapping analysis, protein abundances were transformed to rank normal scores to minimize the effect of outliers (Conover and Iman 1981).

### Age effects on transcript expression

We used the DESeq2 package (Love et al. 2014) to test for transcripts whose expression changed with age. Briefly, we fit the following generalized linear model (GLM) using the link function for the negative binomial distribution in DESeq2:

(2) $$\mathrm{Transcrip}t_{i}=\mathrm{Sex}[i]+\mathrm{Gen}[i]+\mathrm{Age}[i]$$

where Transcript_*i*_ is the total count for each transcript from mouse *i*, Sex[*i*] is the effect corresponding to the sex of mouse *i*, Gen[*i*] is the effect corresponding to the generation of mouse *i*, and Age[*i*] is the effect corresponding to the age of mouse *i*, fit as a continuous variable at the year scale (0.5, 1, 1.5 yr) obtained by dividing the age groups by 12. The age effect represents an estimate of the log_2_ fold change per year of life and was tested using a likelihood ratio test. Transcripts with a significant age effect on expression were determined after FDR adjustment to account for multiple testing across all transcripts (FDR < 0.01).

### Age effects on protein abundance

To detect proteins that change in abundance with age, we fit a log-normal linear model with predictors similar to Equation 2:

(3) $$\mathrm{Protei}n_{i}=\mathrm{Sex}[i]+\mathrm{Gen}[i]+\mathrm{Age}[i]+\varepsilon_{i}$$

where Protein_*i*_ is the log-scale abundance of each protein from mouse *i*, as defined in Equation 1, ɛ_*i*_ is the residual, and all other terms as previously defined. The age effect corresponds to the slope of the regression model and is an estimate of the log_2_ fold change per year of life, which we tested through ANOVA. Proteins with significant age effects were identified after FDR adjustment (FDR < 0.01).

### Age effects on the correlation among protein complex members

To investigate coregulatory patterns of protein complexes (Giurgiu et al. 2019) (CORUM database) and their implications on stoichiometry, we adapted a method described in McKenzie et al. (2016). We filtered the data to only include proteins from only complexes represented by at least four members in our data. We computed Pearson's correlations between all observed gene pairs in the protein complexes at both transcript and protein levels, stratified by age group. Then for each protein complex and data type (transcript and protein), we regressed the correlation coefficients of each gene pair on age and recorded the slope, which represents the change in units of correlation per year:

(4) $${\mathrm{Correlation}}_{k}^{i,j}=\mu +Age[k]+\varepsilon_{k}$$

where ${\mathrm{Correlation}}_{k}^{i,j}$ is the correlation between transcripts or proteins *i* and *j* at age *k*; *μ* is the overall intercept; *Age* is the age effect, fit from a continuous encoding of age at the year scale; $Age[k]={\text{β}}_{Age}*k$; and ɛ_*k*_ is the residual at age *k*. To determine significance, we shuffled the mouse IDs and repeated the slope estimation 1000 times and obtained FDR estimates using the DGCA package (McKenzie et al. 2016). Significant age effects were declared at FDR < 0.1.

We also jointly modeled all gene pairs for each protein complex to estimate an overall age effect instead of fitting separate models per gene pair. We did this by fitting a LMM using the lme4 package to model gene pairs with a random effect, allowing the intercept and age slope to vary with gene pair:

(5) $$\mathrm{Correlatio}n_{ijk}=\mu +u[ij]+({\text{β}}_{\mathrm{Age}}+v_{\mathrm{Age}}[ij])x_{k}+\varepsilon_{ijk}$$

where Correlation_*ijk*_ is the correlation between proteins *i* and *j* at age *k*, *μ* is the overall intercept, *u*[*ij*] is the random deviation on the intercept specific to the pairing of transcripts or proteins *i* and *j*, β_Age_ is the overall age effect, *v*_Age_[*ij*] is the random deviation on the age effect specific to the paring of transcripts or proteins *i* and *j*, *x*_*k*_ is the age (6, 12, or 18 mo), and ɛ_*ijk*_ is random noise on the correlation for proteins *i* and *j* at age *x*_*k*_. The gene pair–specific random terms are modeled as $u∼N(0,\;I\tau^{2})$ and $v_{\mathrm{Age}}\;∼N(0,\;I\tau_{\mathrm{Age}}^{2})$, and the error as $\varepsilon_{ijk}\;∼N(0,\;I\sigma^{2})$. We used the permutations procedure from McKenzie et al. (2016) to determine significance, using a *P*-value cutoff of 0.05, for each protein complex.

### Additive QTL mapping

Although the results of additive QTL mapping are not reported here, we do make them available along with the data, and it is useful to describe their method, which acts as foundation for the age-interactive QTL analysis. For each transcript or protein, we transformed the data to rank normal scores (Conover and Iman 1981) and fit the following model at approximately 64,000 equally spaced loci across the genome:

(6) $$y_{i}=\;\mathrm{QT}L_{m}\left[i\right]+\mathrm{Sex}[i]+\mathrm{Age}[i]+u[mi]+\varepsilon {\;}_{i}$$

where y_*i*_ is the transcript expression or protein abundance (for the gene being analyzed) for mouse *i*, QTL_*m*_[i] is the effect of the founder haplotype at locus *m* (based on expected additive dosages) for mouse *i*, and *u*[*mi*] is a random kinship effect that accounts for the correlation between individual DO mice owing to shared genetic effects excluding the chromosome of locus *m* (leave-one-chromosome-out, i.e., LOCO method). The kinship effect is modeled as $u[m]∼N(0,\;K_{c[m]}K^{2})$, where **K**_*c*[*m*]_ is the realized genomic relationship matrix excluding markers from chromosome *c* of marker *m*, and $\tau_{K}^{2}$ is the variance component underlying the kinship effect (Yang et al. 2014). The log_10_ likelihood ratio (LOD score) was determined by comparing the QTL model (Equation 6) to the null model without the QTL term.

### Age-interactive QTL mapping

We performed a second set of genome scans to identify age-interactive QTL where the rate of change with age of a transcript or protein is dependent on genotype. Genome scans for age-QTL are based on the following model:

(7) $$y_{i}=\;\mathrm{QT}L_{m\left[i\right]\times Age[i]}+\mathrm{QT}L_{m}\left[i\right]+\mathrm{Sex}[i]+\mathrm{Age}[i]+u[mi]+\varepsilon_{i}$$

where QTL_*m*[*i*]_ × *Age*[*i*] is the interaction effect between the QTL genotype and age of mouse *i*. All other terms are as previously defined. The null model for the age-interactive genome scans is the additive QTL model from Equation 6, thus only the interaction term is being tested. To determine significance thresholds for age-QTL, we performed a more elaborate permutation procedure than the standard used for additive QTL (Churchill and Doerge 1994). For each transcript or protein, we fit the following model:

(8) $$y_{i}=\;\mathrm{Sex}[i]+\mathrm{Age}[i]+u_{i}+\varepsilon_{i}.$$

where the kinship term *u*_*i*_ includes effects of all loci, including the additive effect of the locus under evaluation (non-LOCO). We then computed the residuals by subtracting the fitted values of model predictors:

(9) $$e_{i}=\;y_{i}\;-\;\hat{\mathrm{Sex}[i]}+\hat{\mathrm{Age}[i]}+\hat{u_{i}}$$

To construct a permutation test for the age-by-QTL effect, we generate null data by summing the fitted effect values with permutations of the residuals estimated in Equation 7. We repeated the age-interactive scans on the residual-permuted phenotypes 1000 times to obtain a null distribution of the LOD_int_ statistic. Significance thresholds to control the genome-wide error rate at <0.05 for the maximum LOD_int_ scores were based on the 95th percentile of this distribution, resulting in a threshold of LOD_int_ > 7.75. We also defined suggestive age-QTL for transcripts and proteins based on LOD_int_ > 6.0, corresponding to the 37th percentile of the permutation distribution. All QTL analyses were performed with the R/qtl2 package (Broman et al. 2019).

### Distal QTL hotspot analysis

Using a sliding window of 4 Mb, we estimated the density of suggestive age-QTL at positions spanning the genome (LOD_int_ > 6) for transcripts and proteins (Fig. 5A). We defined a genomic region as a hotspot based on mapping more than 30 age-QTL. We used the hotspots to defines sets of transcripts and proteins for functional enrichment analysis. We further refined the hotspot sets by filtering out transcripts or proteins with a mean Pearson correlation coefficient < 0.3 with the other hotspot members, because these genes are less likely to share genetic drivers.

### Functional enrichment analysis

We performed functional enrichment analysis for gene sets defined by differential expression analysis based on age for transcripts and proteins as well as sets defined by the refined age-QTL hotspots. For the sets defined by age effects, we used the R package FGSEA (Korotkevich et al. 2016), whose input is not based on sets defined by significance cutoffs and instead incorporates information from a specified score. We used the standardized age effects from transcripts and proteins as the score, thus up-weighting the influence of genes with more extreme age effects on the enrichment analysis. We considered enrichment categories representing biological process, cellular compartment, and molecular function. We filtered out enrichment results based on a FDR < 0.05 and used a built in function from FGSEA to collapse redundant categories. For the enrichment analysis of gene sets defined by distal age-pQTLs hotspots, we used the clusterProfiler package (Yu et al. 2012), because there is no clear score to incorporate into the analysis. Similarly to the enrichment analysis for age effects, we also considered GO terms for biological processes, cellular compartments, and molecular function for each set and used FDR < 0.05 cutoff to define enriched categories.

### Software

All data analyses and figures were generated using R v4.1.0 (R Core Team 2021) based on the main packages tidyverse v1.3.1, DESeq2 v1.32.0, fgsea v1.18.0, msigdbr v7.4.1, qtl2 v0.24, ensimplR v0.3.0, corrplot 0.90, DGCA v1.0.2, grid v4.1.0, gridExtra v2.3, and stringr v1.4.0. The R Scripts used for all the data processing and analysis can be found on Figshare (https://doi.org/10.6084/m9.figshare.12378077.v6) and as Supplemental Code along with a PDF file describing the workflow.

## Data access

All raw sequencing data generated in this study have been submitted to the NCBI BioProject database (https://www.ncbi.nlm.nih.gov/bioproject/) under accession number PRJNA510989. All the mass spectrometry data generated here have been submitted to the ProteomeXchange database (http://www.proteomexchange.org/) under accession number PXD023724. Both raw and normalized expression and abundance matrices data have been submitted to Figshare database (https://doi.org/10.6084/m9.figshare.12378077.v6). All genotype data for the mice used in this study have been submitted to the DOdb database (https://dodb.jax.org/) under “Shock Center Longitudinal Study” project. All the analyses were performed using as input a compressed RData object containing the processed data and QTL mapping results (JAC_DO_heart_v9.gz), which can be found on Figshare (10.6084/m9.figshare.12378077). These data are also available on QTL Viewer (https://qtlviewer.jax.org/viewer/agingheart) for both interactive analysis and download. If using the download option, users will have access to the same data in the RData object when loading all files (core.JAC_DO_heart.v9.Rdata, dataset.mrna.JAC_DO_heart.v9.Rds, and dataset.protein.JAC_ DO_heart.v9.Rds) into R.

## Supplementary Material

Supplemental Material

## References

[GR275672GERC1] Albert FW, Bloom JS, Siegel J, Day L, Kruglyak L. 2018. Genetics of *trans*-regulatory variation in gene expression. eLife 7: 7. 10.7554/eLife.35471 PMC6072440 30014850

[GR275672GERC2] Anisimova AS, Alexandrov AI, Makarova NE, Gladyshev VN, Dmitriev SE. 2018. Protein synthesis and quality control in aging. Aging 10: 4269–4288. 10.18632/aging.101721 30562164 PMC6326689

[GR275672GERC4] Avila JJ, Kim SK, Massett MP. 2017. Differences in exercise capacity and responses to training in 24 inbred mouse strains. Front Physiol 8: 974. 10.3389/fphys.2017.00974 29249981 PMC5714923

[GR275672GERC5] Bahar R, Hartmann CH, Rodriguez KA, Denny AD, Busuttil RA, Dollé MET, Calder RB, Chisholm GB, Pollock BH, Klein CA, 2006. Increased cell-to-cell variation in gene expression in ageing mouse heart. Nature 441: 1011–1014. 10.1038/nature04844 16791200

[GR275672GERC6] Barrick CJ, Rojas M, Schoonhoven R, Smyth SS, Threadgill DW. 2007. Cardiac response to pressure overload in 129S1/SvImJ and C57BL/6J mice: temporal- and background-dependent development of concentric left ventricular hypertrophy. Am J Physiol Heart Circ Physiol 292: H2119–H2130. 10.1152/ajpheart.00816.2006 17172276

[GR275672GERC7] Bartling B, Niemann K, Pliquett RU, Treede H, Simm A. 2019. Altered gene expression pattern indicates the differential regulation of the immune response system as an important factor in cardiac aging. Exp Gerontol 117: 13–20. 10.1016/j.exger.2018.05.001 29738791

[GR275672GERC8] Basler M, Kirk CJ, Groettrup M. 2013. The immunoproteasome in antigen processing and other immunological functions. Curr Opin Immunol 25: 74–80. 10.1016/j.coi.2012.11.004 23219269

[GR275672GERC9] Bates D, Mächler M, Bolker B, Walker S. 2015. Fitting linear mixed-effects models using *lme4*. J Stat Softw 67: 1. 10.18637/jss.v067.i01

[GR275672GERC10] Battle A, Khan Z, Wang SH, Mitrano A, Ford MJ, Pritchard JK, Gilad Y. 2015. Impact of regulatory variation from RNA to protein. Science 347: 664–667. 10.1126/science.1260793 25657249 PMC4507520

[GR275672GERC11] Berger J, Berger S, Li M, Jacoby AS, Arner A, Bavi N, Stewart AG, Currie PD. 2018. *In vivo* function of the chaperonin TRiC in α-actin folding during sarcomere assembly. Cell Rep 22: 313–322. 10.1016/j.celrep.2017.12.069 29320728

[GR275672GERC12] Broman KW, Gatti DM, Simecek P, Furlotte NA, Prins P, Sen Ś, Yandell BS, Churchill GA. 2019. R/qtl2: software for mapping quantitative trait loci with high-dimensional data and multiparent populations. Genetics 211: 495–502. 10.1534/genetics.118.301595 30591514 PMC6366910

[GR275672GERC13] Bucci C, Parton RG, Mather IH, Stunnenberg H, Simons K, Hoflack B, Zerial M. 1992. The small GTPase rab5 functions as a regulatory factor in the early endocytic pathway. Cell 70: 715–728. 10.1016/0092-8674(92)90306-W 1516130

[GR275672GERC15] Carrard G, Bulteau AL, Petropoulos I, Friguet B. 2002. Impairment of proteasome structure and function in aging. Int J Biochem Cell Biol 34: 1461–1474. 10.1016/S1357-2725(02)00085-7 12200039

[GR275672GERC16] Cellerino A, Ori A. 2017. What have we learned on aging from omics studies? Sem Cell Dev Biol 70: 177–189. 10.1016/j.semcdb.2017.06.012 28630026

[GR275672GERC17] Chiao YA, Rabinovitch PS. 2015. The aging heart. Cold Spring Harb Perspect Med 5: a025148. 10.1101/cshperspect.a025148 26328932 PMC4561390

[GR275672GERC18] Chick JM, Munger SC, Simecek P, Huttlin EL, Choi K, Daniel M. 2016. Defining the consequences of genetic variation on a proteome-wide scale. Nature 534: 500–505. 10.1038/nature18270 27309819 PMC5292866

[GR275672GERC19] Churchill GA, Doerge RW. 1994. Empirical threshold values for quantitative trait mapping. Genetics 138: 963–971. 10.1093/genetics/138.3.963 7851788 PMC1206241

[GR275672GERC20] Collard F, Delpierre G, Stroobant V, Matthijs G, Van Schaftingen E. 2003. A mammalian protein homologous to fructosamine-3-kinase is a ketosamine-3-kinase acting on psicosamines and ribulosamines but not on fructosamines. Diabetes 52: 2888–2895. 10.2337/diabetes.52.12.2888 14633848

[GR275672GERC21] Conover WJ, Iman RL. 1981. Rank transformations as a bridge between parametric and nonparametric statistics. Am Stat 35: 124. 10.2307/2683975

[GR275672GERC22] Conti A, Gorza L, Sorrentino V. 1996. Differential distribution of ryanodine receptor type 3 (RyR3) gene product in mammalian skeletal muscles. Biochem J 316: 19. 10.1042/bj3160019 8645204 PMC1217321

[GR275672GERC23] Coux O, Tanaka K, Goldberg AL. 1996. Structure and functions of the 20S and 26S proteasomes. Annu Rev Biochem 65: 801–847. 10.1146/annurev.bi.65.070196.004101 8811196

[GR275672GERC24] Dai DF, Chen T, Johnson SC, Szeto H, Rabinovitch PS. 2012. Cardiac aging: from molecular mechanisms to significance in human health and disease. Antioxid Redox Signal 16: 1492–1526. 10.1089/ars.2011.4179 22229339 PMC3329953

[GR275672GERC25] D'Angelo MA, Raices M, Panowski SH, Hetzer MW. 2009. Age-dependent deterioration of nuclear pore complexes causes a loss of nuclear integrity in postmitotic cells. Cell 136: 284–295. 10.1016/j.cell.2008.11.037 19167330 PMC2805151

[GR275672GERC26] Eng JK, McCormack AL, Yates JR. 1994. An approach to correlate tandem mass spectral data of peptides with amino acid sequences in a protein database. J Am Soc Mass Spectrom 5: 976–989. 10.1016/1044-0305(94)80016-2 24226387

[GR275672GERC27] Forte E, Furtado MB, Rosenthal N. 2018. The interstitium in cardiac repair: role of the immune-stromal cell interplay. Nat Rev Cardiol 15: 601–616. 10.1038/s41569-018-0077-x 30181596

[GR275672GERC28] Forte E, Skelly DA, Chen M, Daigle S, Morelli KA, Hon O, Philip VM, Costa MW, Rosenthal NA, Furtado MB. 2020. Dynamic interstitial cell response during myocardial infarction predicts resilience to rupture in genetically diverse mice. Cell Rep 30: 3149–3163.e6. 10.1016/j.celrep.2020.02.008 32130914 PMC7059115

[GR275672GERC29] Gatti DM, Svenson KL, Shabalin A, Wu LY, Valdar WW, Simecek P, Goodwin N, Cheng R, Pomp D, Palmer A, 2014. Quantitative trait locus mapping methods for diversity outbred mice. G3 (Bethesda) 4: 1623–1633. 10.1534/g3.114.013748 25237114 PMC4169154

[GR275672GERC14] The Gene Ontology Consortium. 2021. The Gene Ontology resource: enriching a GOld mine. Nucleic Acids Res 49: D325–D334. 10.1093/nar/gkaa1113 33290552 PMC7779012

[GR275672GERC3] The Gene Ontology Consortium, Ashburner M, Ball CA, Blake JA, Botstein D, Butler H, Cherry JM, Davis AP, Dolinski K, Dwight SS, 2000. Gene Ontology: tool for the unification of biology. Nat Genet 25: 25–29. 10.1038/75556 10802651 PMC3037419

[GR275672GERC30] Giurgiu M, Reinhard J, Brauner B, Dunger-Kaltenbach I, Fobo G, Frishman G, Montrone C, Ruepp A. 2019. CORUM: the comprehensive resource of mammalian protein complexes—2019. Nucleic Acids Res 47: D559–D563. 10.1093/nar/gky973 30357367 PMC6323970

[GR275672GERC31] Gonskikh Y, Polacek N. 2017. Alterations of the translation apparatus during aging and stress response. Mech Ageing Dev 168: 30–36. 10.1016/j.mad.2017.04.003 28414025

[GR275672GERC32] Greenig M, Melville A, Huntley D, Isalan M, Mielcarek M. 2020. Cross-sectional transcriptional analysis of the aging murine heart. Front Mol Biosci 7: 237. 10.3389/fmolb.2020.565530 PMC7545256 33102519

[GR275672GERC33] Han J, Lee Y, Yeom KH, Kim YK, Jin H, Kim VN. 2004. The Drosha-DGCR8 complex in primary microRNA processing. Genes Dev 18: 3016. 10.1101/gad.1262504 15574589 PMC535913

[GR275672GERC34] Hause RJ, Stark AL, Antao NN, Gorsic LK, Chung SH, Brown CD, Wong SS, Gill DF, Myers JL, To LA, 2014. Identification and validation of genetic variants that influence transcription factor and cell signaling protein levels. Am J Hum Genet 95: 194–208. 10.1016/j.ajhg.2014.07.005 25087611 PMC4129400

[GR275672GERC35] Higuchi-Sanabria R, Frankino PA, Paul JW, Tronnes SU, Dillin A. 2018. A futile battle? Protein quality control and the stress of aging. Dev Cell 44: 139–163. 10.1016/j.devcel.2017.12.020 29401418 PMC5896312

[GR275672GERC36] Holm S. 1979. A simple sequentially rejective multiple test procedure a simple sequentially rejective multiple test procedure. Scand J Statist 6: 65–70.

[GR275672GERC37] Huttlin EL, Jedrychowski MP, Elias JE, Goswami T, Rad R, Beausoleil SA, Villén J, Haas W, Sowa ME, Gygi SP. 2010. A tissue-specific atlas of mouse protein phosphorylation and expression. Cell 143: 1174–1189. 10.1016/j.cell.2010.12.001 21183079 PMC3035969

[GR275672GERC38] Işıldak U, Somel M, Thornton JM, Dönertaş HM. 2020. Temporal changes in the gene expression heterogeneity during brain development and aging. Sci Rep 10: 4080. 10.1038/s41598-020-60998-0 32139741 PMC7058021

[GR275672GERC39] Johnson AA, Shokhirev MN, Wyss-Coray T, Lehallier B. 2020. Systematic review and analysis of human proteomics aging studies unveils a novel proteomic aging clock and identifies key processes that change with age. Ageing Res Rev 60: 101070. 10.1016/j.arr.2020.101070 32311500

[GR275672GERC40] Kajiho H, Sakurai K, Minoda T, Yoshikawa M, Nakagawa S, Fukushima S, Kontani K, Katada T. 2011. Characterization of RIN3 as a guanine nucleotide exchange factor for the Rab5 subfamily GTPase Rab31. J Biol Chem 286: 24364. 10.1074/jbc.M110.172445 21586568 PMC3129215

[GR275672GERC41] Kang MJ, Yang S, Baek JW, Shim YS, Oh YJ, Hwang IT. 2017. Fetuin-A as an alternative marker for insulin resistance and cardiovascular risk in prepubertal children. J Atheroscler Thromb 24: 1031–1038. 10.5551/jat.38323 28154244 PMC5656765

[GR275672GERC42] Keele GR, Zhang T, Pham DT, Vincent M, Bell TA, Hock P, Shaw GD, Paulo JA, Munger SC, Pardo-Manuel de Villena F, 2021. Regulation of protein abundance in genetically diverse mouse populations. Cell Genomics 1: 100003. 10.1016/j.xgen.2021.100003 PMC9536773 36212994

[GR275672GERC43] Kelmer Sacramento E, Kirkpatrick JM, Mazzetto M, Baumgart M, Bartolome A, Di Sanzo S, Caterino C, Sanguanini M, Papaevgeniou N, Lefaki M, 2020. Reduced proteasome activity in the aging brain results in ribosome stoichiometry loss and aggregation. Mol Syst Biol 16: e9596. 10.15252/msb.20209596 32558274 PMC7301280

[GR275672GERC44] Kiper C, Grimes B, Van Zant G, Satin J. 2013. Mouse strain determines cardiac growth potential. PLoS One 8: e70512. 10.1371/journal.pone.0070512 23940585 PMC3734269

[GR275672GERC45] Korotkevich G, Sukhov V, Budin N, Shpak B, Artyomov M, Sergushichev A. 2016. An algorithm for fast preranked gene set enrichment analysis using cumulative statistic calculation. bioRxiv 10.1101/060012

[GR275672GERC46] Lakatta EG, Levy D. 2003. Arterial and cardiac aging: major shareholders in cardiovascular disease enterprises. Circulation 107: 139–146. 10.1161/01.CIR.0000048892.83521.58 12515756

[GR275672GERC47] Li YF, Wang X. 2011. The role of the proteasome in heart disease. Biochim Biophys Acta 1809: 141. 10.1016/j.bbagrm.2010.09.001 20840877 PMC3021001

[GR275672GERC48] López-Otín C, Blasco MA, Partridge L, Serrano M, Kroemer G. 2013. The hallmarks of aging. Cell 153: 1194. 10.1016/j.cell.2013.05.039 23746838 PMC3836174

[GR275672GERC49] Love MI, Huber W, Anders S. 2014. Moderated estimation of fold change and dispersion for RNA-seq data with DESeq2. Genome Biol 15: 550. 10.1186/s13059-014-0550-8 25516281 PMC4302049

[GR275672GERC50] Martinez O, Goud B. 1998. Rab proteins. Biochim Biophys Acta Mol Cell Res 1404: 101–112. 10.1016/S0167-4889(98)00050-0 9714762

[GR275672GERC51] McKenzie AT, Katsyv I, Song WM, Wang M, Zhang B. 2016. DGCA: a comprehensive R package for Differential Gene Correlation Analysis. BMC Syst Biol 10: 106. 10.1186/s12918-016-0349-1 27846853 PMC5111277

[GR275672GERC52] Melzer D, Hurst AJ, Frayling T. 2007. Genetic variation and human aging: progress and prospects. J Gerontol A Biol Sci Med Sci 62: 301–307. 10.1093/gerona/62.3.301 17389728

[GR275672GERC53] Moro-García MA, Alonso-Arias R, López-Larrea C. 2013. When aging reaches CD4+ T-cells: phenotypic and functional changes. Front Immunol 4: 107. 10.3389/fimmu.2013.00107 23675374 PMC3650461

[GR275672GERC54] Müller-Werdan U. 2007. Inflammation and ageing. Z Gerontol Geriatr 40: 362–365. 10.1007/s00391-007-0486-7 17943240

[GR275672GERC55] Murshid A, Eguchi T, Calderwood SK. 2013. Stress proteins in aging and life span. Int J Hyperthermia 29: 442. 10.3109/02656736.2013.798873 23742046 PMC4083487

[GR275672GERC56] Nakai A, Yamaguchi O, Takeda T, Higuchi Y, Hikoso S, Taniike M, Omiya S, Mizote I, Matsumura Y, Asahi M, 2007. The role of autophagy in cardiomyocytes in the basal state and in response to hemodynamic stress. Nat Med 13: 619–624. 10.1038/nm1574 17450150

[GR275672GERC57] North BJ, Sinclair DA. 2012. The intersection between aging and cardiovascular disease. Circ Res 110: 1097–1108. 10.1161/CIRCRESAHA.111.246876 22499900 PMC3366686

[GR275672GERC58] Ori A, Toyama BH, Harris MS, Bock T, Iskar M, Bork P, Ingolia NT, Hetzer MW, Beck M. 2015. Integrated transcriptome and proteome analyses reveal organ-specific proteome deterioration in old rats. Cell Syst 1: 224–237. 10.1016/j.cels.2015.08.012 27135913 PMC4802414

[GR275672GERC59] Ori A, Iskar M, Buczak K, Kastritis P, Parca L, Andrés-Pons A, Singer S, Bork P, Beck M. 2016. Spatiotemporal variation of mammalian protein complex stoichiometries. Genome Biol 17: 47. 10.1186/s13059-016-0912-5 26975353 PMC4791834

[GR275672GERC60] Palombella VJ, Rando OJ, Goldberg AL, Maniatis T. 1994. The ubiquitin-proteasome pathway is required for processing the NF-κB1 precursor protein and the activation of NF-κB. Cell 78: 773–785. 10.1016/S0092-8674(94)90482-0 8087845

[GR275672GERC61] Pillai VB, Sundaresan NR, Gupta MP. 2014. Regulation of Akt signaling by sirtuins: its implication in cardiac hypertrophy and aging. Circ Res 114: 368–378. 10.1161/CIRCRESAHA.113.300536 24436432 PMC4228987

[GR275672GERC62] Predmore JM, Wang P, Davis F, Bartolone S, Westfall MV, Dyke DB, Pagani F, Powell SR, Day SM. 2010. Ubiquitin proteasome dysfunction in human hypertrophic and dilated cardiomyopathies. Circulation 121: 997–1004. 10.1161/CIRCULATIONAHA.109.904557 20159828 PMC2857348

[GR275672GERC63] Quarles EK, Dai DF, Tocchi A, Basisty N, Gitari L, Rabinovitch PS. 2015. Quality control systems in cardiac aging. Ageing Res Rev 23: 101–115. 10.1016/j.arr.2015.02.003 25702865 PMC4686341

[GR275672GERC64] Raghupathy N, Choi K, Vincent MJ, Beane GL, Sheppard KS, Munger SC, Korstanje R, Pardo-Manual de Villena F, Churchill GA. 2018. Hierarchical analysis of RNA-seq reads improves the accuracy of allele-specific expression. Bioinformatics 34: 2177–2184. 10.1093/bioinformatics/bty078 29444201 PMC6022640

[GR275672GERC65] Ramosa GC, Van Den Berg A, Nunes-Silva V, Weirather J, Peters L, Burkard M, Friedrich M, Pinnecker J, Abeer M, Heinzed KG, 2017. Myocardial aging as a T-cell-mediated phenomenon. Proc Natl Acad Sci 114: E2420–E2429. 10.1073/pnas.1621047114 28255084 PMC5373357

[GR275672GERC66] R Core Team. 2021. R: a language and environment for statistical computing. R Foundation for Statistical Computing, Vienna. https://www.R-project.org/.

[GR275672GERC67] Rizvi F, Preston CC, Emelyanova L, Yousufuddin M, Viqar M, Dakwar O, Ross GR, Faustino RS, Holmuhamedov EL, Jahangir A. 2021. Effects of aging on cardiac oxidative stress and transcriptional changes in pathways of reactive oxygen species generation and clearance. J Am Heart Assoc 10: 19948. 10.1161/JAHA.120.019948 PMC8475058 34369184

[GR275672GERC68] Salimova E, Nowak KJ, Estrada AC, Furtado MB, McNamara E, Nguyen Q, Balmer L, Preuss C, Holmes JW, Ramialison M, 2019. Variable outcomes of human heart attack recapitulated in genetically diverse mice. NPJ Regen Med 4: 5. 10.1038/s41536-019-0067-6 30854227 PMC6399323

[GR275672GERC69] Santos-Lozano A, Valenzuela PL, Llavero F, Lista S, Carrera-Bastos P, Hampel H, Pareja-Galeano H, Gálvez BG, López JA, Vázquez J, 2020. Successful aging: insights from proteome analyses of healthy centenarians. Aging 12: 3502–3515. 10.18632/aging.102826 32100723 PMC7066932

[GR275672GERC70] Saul MC, Philip VM, Reinholdt LG, Chesler EJ. 2019. High-diversity mouse populations for complex traits. Trends Genet 35: 501–514. 10.1016/j.tig.2019.04.003 31133439 PMC6571031

[GR275672GERC71] Schaum N, Lehallier B, Hahn O, Pálovics R, Hosseinzadeh S, Lee SE, Sit R, Lee DP, Losada PM, Zardeneta ME, 2020. Ageing hallmarks exhibit organ-specific temporal signatures. Nature 583: 596. 10.1038/s41586-020-2499-y 32669715 PMC7757734

[GR275672GERC72] Shorter JR, Huang W, Beak JY, Hua K, Gatti DM, de Villena FPM, Pomp D, Jensen BC. 2018. Quantitative trait mapping in diversity outbred mice identifies two genomic regions associated with heart size. Mamm Genome 29: 80–89. 10.1007/s00335-017-9730-7 29279960 PMC6340297

[GR275672GERC73] Singh PP, Demmitt BA, Nath RD, Brunet A. 2019. The genetics of aging: a vertebrate perspective. Cell 177: 200–220. 10.1016/j.cell.2019.02.038 30901541 PMC7592626

[GR275672GERC74] Stanley WC, Recchia FA, Lopaschuk GD. 2005. Myocardial substrate metabolism in the normal and failing heart. Physiol Rev 85: 1093–1129. 10.1152/physrev.00006.2004 15987803

[GR275672GERC75] Strait JB, Lakatta EG. 2012. Aging-associated cardiovascular changes and their relationship to heart failure. Heart Fail Clin 8: 143–164. 10.1016/j.hfc.2011.08.011 22108734 PMC3223374

[GR275672GERC76] Strambio-De-Castillia C, Niepel M, Rout MP. 2010. The nuclear pore complex: bridging nuclear transport and gene regulation. Nat Rev Mol Cell Biol 11: 490–501. 10.1038/nrm2928 20571586

[GR275672GERC77] Subramanian A, Tamayo P, Mootha VK, Mukherjee S, Ebert BL, Gillette MA, Paulovich A, Pomeroy SL, Golub TR, Lander ES, 2005. Gene set enrichment analysis: a knowledge-based approach for interpreting genome-wide expression profiles. Proc Natl Acad Sci 102: 15545–15550. 10.1073/pnas.0506580102 16199517 PMC1239896

[GR275672GERC78] Svenson KL, Gatti DM, Valdar W, Welsh CE, Cheng R, Chesler EJ, Palmer AA, McMillan L, Churchill GA. 2012. High-resolution genetic mapping using the mouse diversity outbred population. Genetics 190: 437–447. 10.1534/genetics.111.132597 22345611 PMC3276626

[GR275672GERC79] Szegezdi E, Logue SE, Gorman AM, Samali A. 2006. Mediators of endoplasmic reticulum stress-induced apoptosis. EMBO Rep 7: 880–885. 10.1038/sj.embor.7400779 16953201 PMC1559676

[GR275672GERC80] Szwergold BS, Bunker RD, Loomes KM. 2011. The physiological substrates of fructosamine-3-kinase-related-protein (FN3KRP) are intermediates of nonenzymatic reactions between biological amines and ketose sugars (fructation products). Med Hypotheses 77: 739–744. 10.1016/j.mehy.2011.07.027 21924559

[GR275672GERC81] Taggart JC, Zauber H, Selbach M, Li GW, McShane E. 2020. Keeping the proportions of protein complex components in check. Cell Syst 10: 125–132. 10.1016/j.cels.2020.01.004 32105631 PMC7195860

[GR275672GERC82] Takemon Y, Chick JM, Gerdes Gyuricza I, Skelly DA, Devuyst O, Gygi SP, Churchill GA, Korstanje R. 2021. Proteomic and transcriptomic profiling reveal different aspects of aging in the kidney. eLife 10: e62585. 10.7554/eLife.62585 33687326 PMC8096428

[GR275672GERC83] Taneike M, Yamaguchi O, Nakai A, Hikoso S, Takeda T, Mizote I, Oka T, Tamai T, Oyabu J, Murakawa T, 2010. Inhibition of autophagy in the heart induces age-related cardiomyopathy. Autophagy 6: 600–606. 10.4161/auto.6.5.11947 20431347

[GR275672GERC84] Toko H, Takahashi H, Kayama Y, Okada S, Minamino T, Terasaki F, Kitaura Y, Komuro I. 2010. ATF6 is important under both pathological and physiological states in the heart. J Mol Cell Cardiol 49: 113–120. 10.1016/j.yjmcc.2010.03.020 20380836

[GR275672GERC85] Torres GG, Nygaard M, Caliebe A, Blanché H, Chantalat S, Galan P, Lieb W, Christiansen L, Deleuze JF, Christensen K, 2021. Exome-wide association study identifies *FN3KRP* and *PGP* as new candidate longevity genes. J Gerontol A Biol Sci Med Sci 76: 786–795. 10.1093/gerona/glab023 33491046 PMC8087267

[GR275672GERC86] Tran DH, Wang ZV. 2019. Glucose metabolism in cardiac hypertrophy and heart failure. J Am Heart Assoc 8: e012673. 10.1161/JAHA.119.012673 31185774 PMC6645632

[GR275672GERC87] Waldera-Lupa DM, Kalfalah F, Florea AM, Sass S, Kruse F, Rieder V, Tigges J, Fritsche E, Krutmann J, Busch H, 2014. Proteome-wide analysis reveals an age-associated cellular phenotype of *in situ* aged human fibroblasts. Aging 6: 856–878. 10.18632/aging.100698 25411231 PMC4247387

[GR275672GERC88] Wei YN, Hu HY, Xie GC, Fu N, Bin NZ, Zeng R, Khaitovich P. 2015. Transcript and protein expression decoupling reveals RNA binding proteins and miRNAs as potential modulators of human aging. Genome Biol 16: 41. 10.1186/s13059-015-0608-2 25853883 PMC4375924

[GR275672GERC89] Wilson Van Voorhis CR, Morgan BL. 2007. Understanding power and rules of thumb for determining sample sizes. Tutor Quant Methods Psychol 3: 43–50. 10.20982/tqmp.03.2.p043

[GR275672GERC90] Wu G, Yussman MG, Barrett TJ, Hahn HS, Osinska H, Hilliard GM, Wang X, Toyokawa T, Yatani A, Lynch RA, 2001. Increased myocardial Rab GTPase expression a consequence and cause of cardiomyopathy. Circ Res 89: 1130–1137. 10.1161/hh2401.100427 11739277

[GR275672GERC91] Xing S, Tsaih SW, Yuan R, Svenson KL, Jorgenson LM, So M, Paigen BJ, Korstanje R. 2009. Genetic influence on electrocardiogram time intervals and heart rate in aging mice. Am J Physiol Heart Circ Physiol 296: H1907–H1913. 10.1152/ajpheart.00681.2008 19395551 PMC2716104

[GR275672GERC92] Yang J, Zaitlen NA, Goddard ME, Visscher PM, Price AL. 2014. Advantages and pitfalls in the application of mixed-model association methods. Nat Genet 46: 100–106. 10.1038/ng.2876 24473328 PMC3989144

[GR275672GERC93] Yao C, Chen G, Song C, Keefe J, Mendelson M, Huan T, Sun BB, Laser A, Maranville JC, Wu H, 2018. Genome-wide mapping of plasma protein QTLs identifies putatively causal genes and pathways for cardiovascular disease. Nat Commun 9: 3268. 10.1038/s41467-018-05512-x 30111768 PMC6093935

[GR275672GERC94] Yu G, Wang LG, Han Y, He QY. 2012. clusterProfiler: an R package for comparing biological themes among gene clusters. OMICS 16: 284–287. 10.1089/omi.2011.0118 22455463 PMC3339379

[GR275672GERC95] Zhang T, Keele GR, Churchill GA, Gygi SP, Paulo JA. 2021. Strain-specific peptide (SSP) interference reference sample: a genetically encoded quality control for isobaric tagging strategies. Anal Chem 93: 5241–5247. 10.1021/acs.analchem.0c05483 33735571 PMC8210951

