# Supplementary material for: Genome-wide transcript and protein analysis highlights the role of protein homeostasis in the aging mouse heart
Source: Genome Res. 2022 May;32(5):838–52. doi: 10.1101/gr.275672.121 (PMC9104701; doi:10.1101/gr.275672.121)
Supplement: Supplemental Material [file supp_32_5_838__DC1.html]

Genome-wide transcript and protein analysis highlights the role of protein homeostasis in the aging mouse heart — Supplemental Material 

# Genome-wide transcript and protein analysis highlights the role of protein homeostasis in the aging mouse heart

## Supplemental Material

- Supplemental\_Data\_S1.xls
- Supplemental\_Data\_S2.xls
- Supplemental\_Data\_S3.xls
- Supplemental\_Data\_S4.xls
- Supplemental\_Data\_S5.xls
- Supplemental\_Data\_S6.xls
- Supplemental\_Data\_S7.xls
- Supplemental\_Data\_S8.xls
- Supplemental\_Data\_S9.xls
- Supplemental\_Data\_S10.xlsx
- Supplemental\_Data\_S11.xls
- Supplemental\_Data\_S12.xls
- Supplemental\_Data\_S13.xls
- Supplemental\_Figures.pdf
- Supplemental\_Scripts.zip
