# Supplementary material for: Genome-wide transcript and protein analysis highlights the role of protein homeostasis in the aging mouse heart
Source: Genome Res. 2022 May;32(5):838–52. doi: 10.1101/gr.275672.121 (PMC9104701; doi:10.1101/gr.275672.121)
Supplement: Supplemental Material [file supp_gr.275672.121_Supplemental_Figures_.pdf]

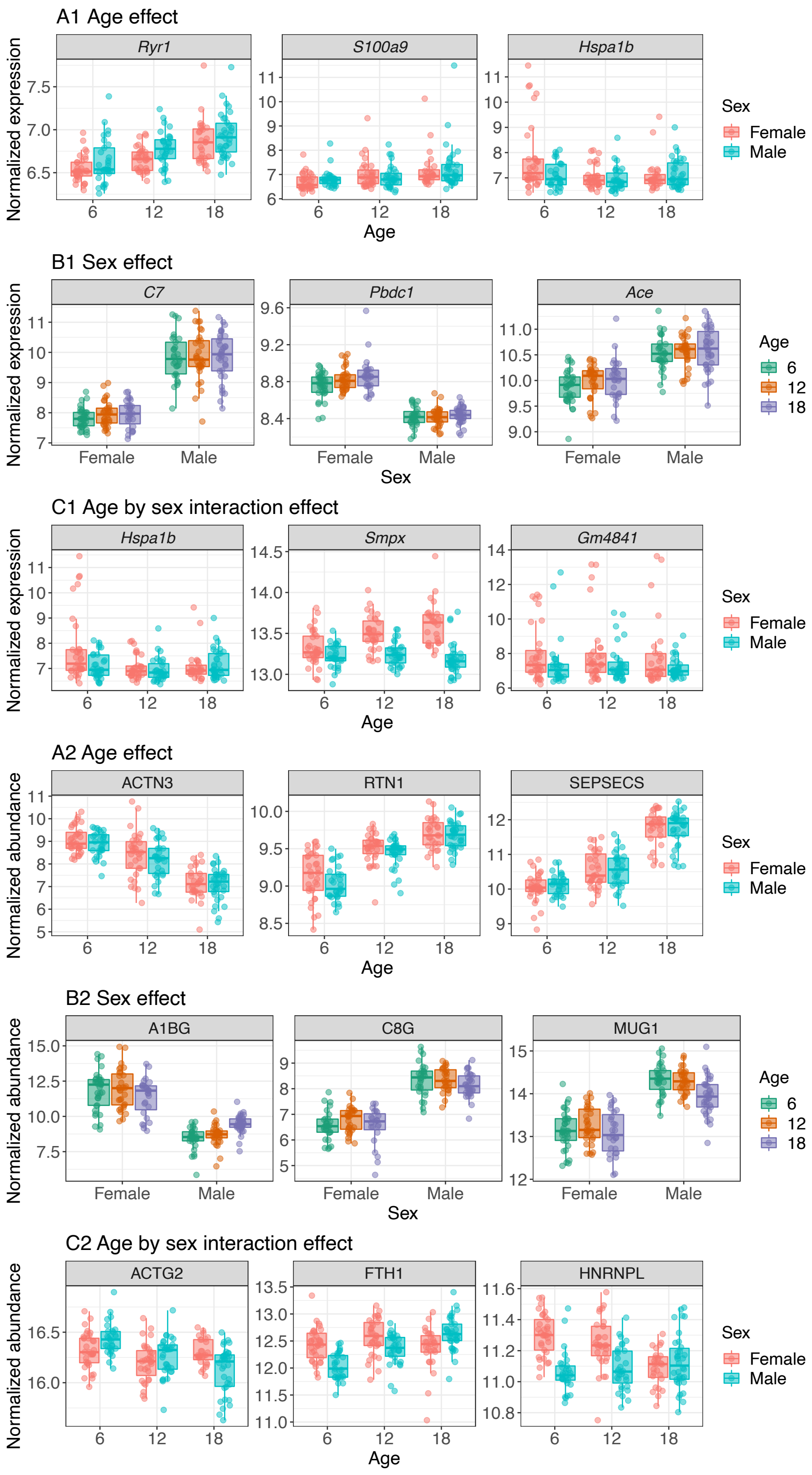

Supplemental Figure S1: Examples of age, sex, and age-by-sex effects on transcripts and proteins. Normalized expression (y-axis) of transcripts (1) or normalized abundance (y-axis) of proteins (2) demonstrating a significant (FDR < 0.01) age effect (A), sex effect (B) and age by sex interaction effect (C). In A) and C) the data are plotted by age on the x-axis and stratified and colored according to sex. In B) the data are plotted by sex on the x-axis and stratified and colored according to age group.

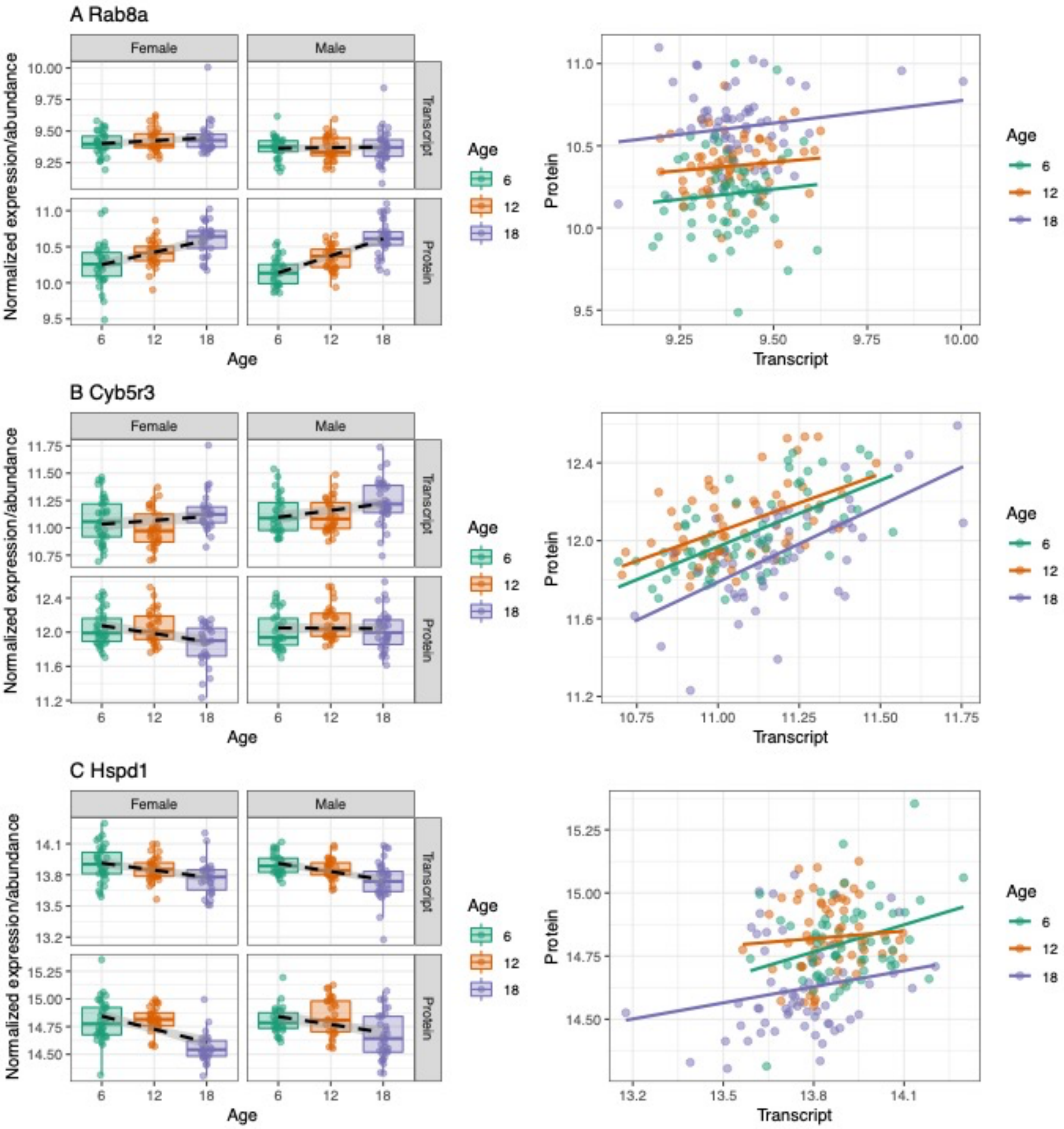

Supplemental Figure S2: Examples of genes with age effects, highlighted through functional enrichment analysis. Normalized (top) gene expression and (bottom) protein abundance (y-axis) by age (x-axis), stratified by sex on the left, and normalized protein abundance (y-axis) by normalized transcript expression (x-axis) on the right for the genes (A) *Rab8a*, (B) *Cyb5r3* and (C) *Hspd1*. Points are colored according to age group. Best fit regression lines for each age group included to highlight trends.

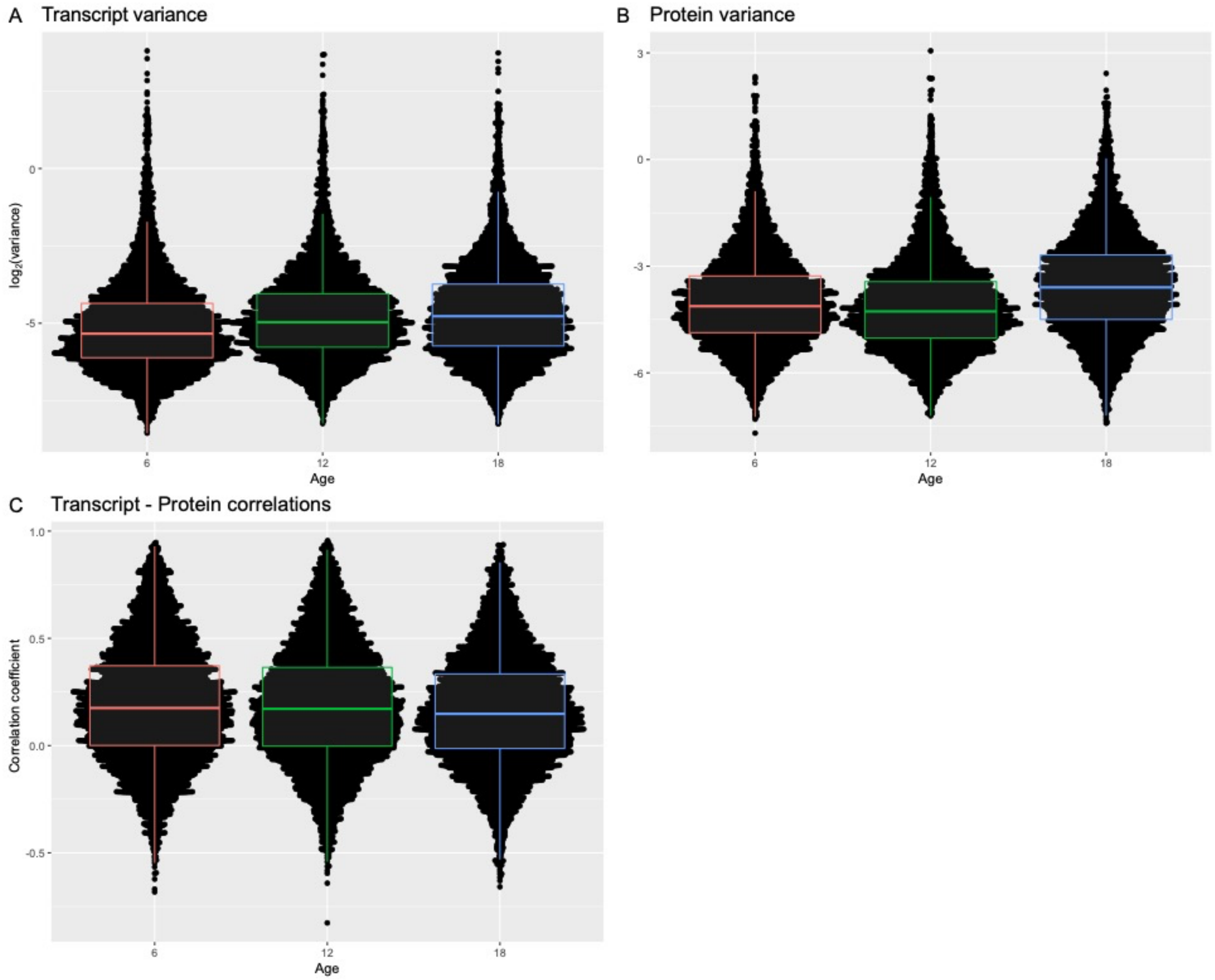

Supplemental Figure S3: Variances and correlation of proteins and transcripts across age groups. Log_2 variances for (A) transcripts and (B) proteins, stratified by age group. Each point represents a transcript or protein. C) Pearson correlation coefficients for transcript-protein pairs, stratified by age group. Each point represents a transcript-protein pair for a given age group.

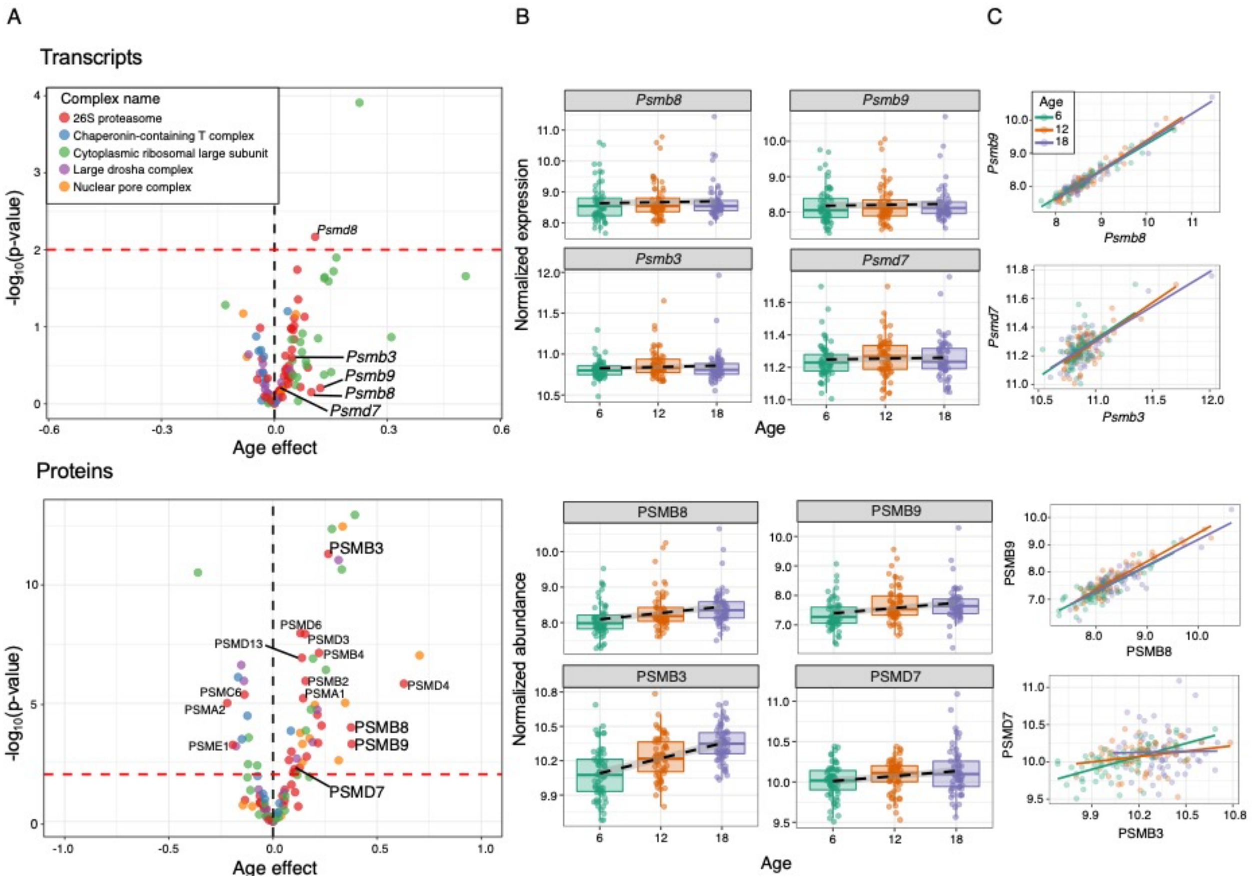

Supplemental Figure S4: Age effects on transcripts and proteins that form protein complexes. A) Volcano plots show the -$\log_{10}$(p-value) (y-axis) by age effects (x-axis) for members of protein complexes at the transcript (top) and protein (bottom) levels. The horizontal red line represents the significance cut-off at FDR < 0.01. Age effects are reported as $\log_2$ change in abundance per year. Included genes represent the 26S proteasome, chaperonin-containing T complex, cytoplasmic ribosomal large subunit, large drosha complex and nuclear pore complex (indicated by colors), which are the protein complexes most affected by age based on correlation. Genes from the 26S proteasome are highlighted. Many members have significant age effects on the proteins, but much less so on transcripts. Vertical line at zero included for reference. B) Normalized abundance (y-axis) for transcripts (top) and proteins (bottom) across age (x-axis) for the genes *Psmb8*, *Psmb9*, *Psmb3* and *Psmd7*. These genes tend to increase with age at both transcript and protein levels. Best fit lines included to highlight trends. C) Correlation between the *Psmb8* (x-axis) and *Psmb9* (y-axis) and the *Psmb3* (x-axis) and *Psmd7* (y-axis) for both transcripts (top) and proteins (bottom) across age groups (indicated by color). The correlation between *Psmb8* and *Psmb9* is maintained across age groups for both transcript and protein. Even though *Psmb3* and *Psmd7* change in the same direction with age, their correlation is reduced with age in proteins.

26S proteasome

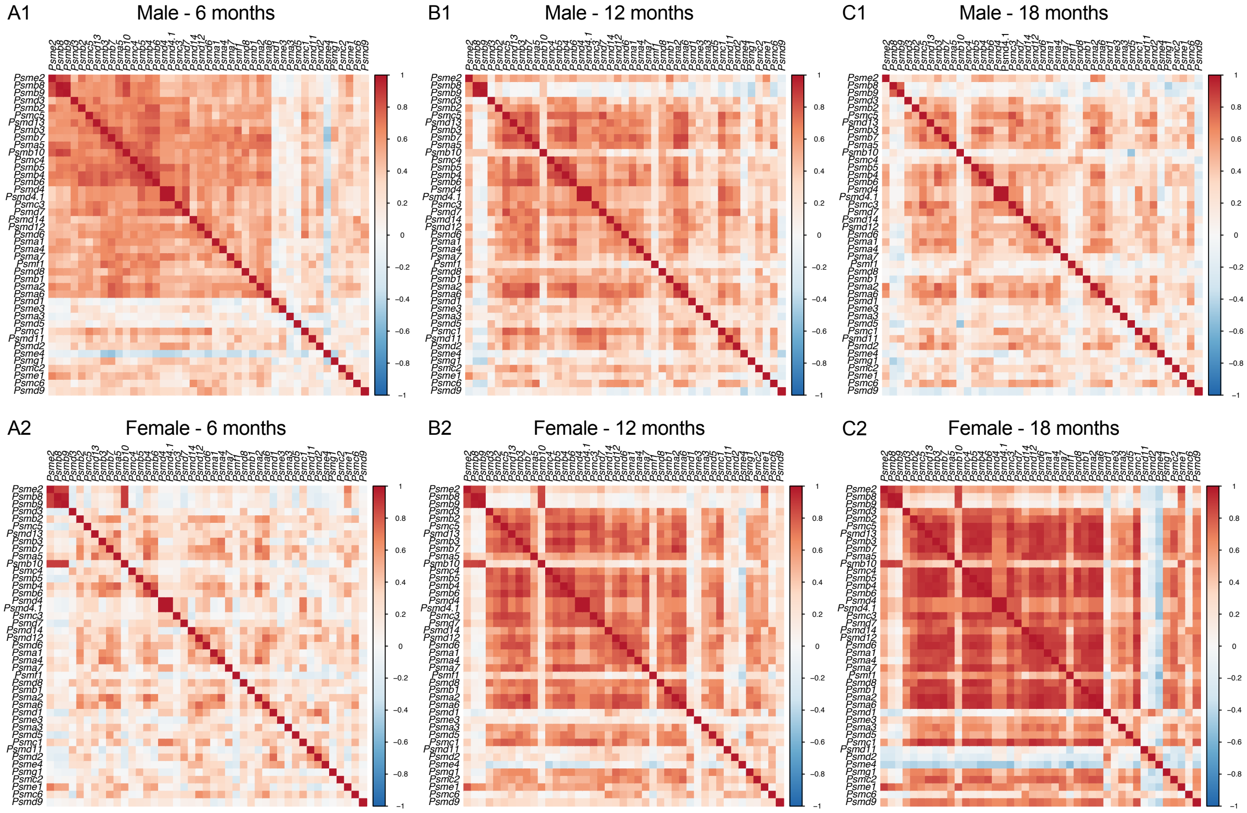

Chaperonin-containing T complex

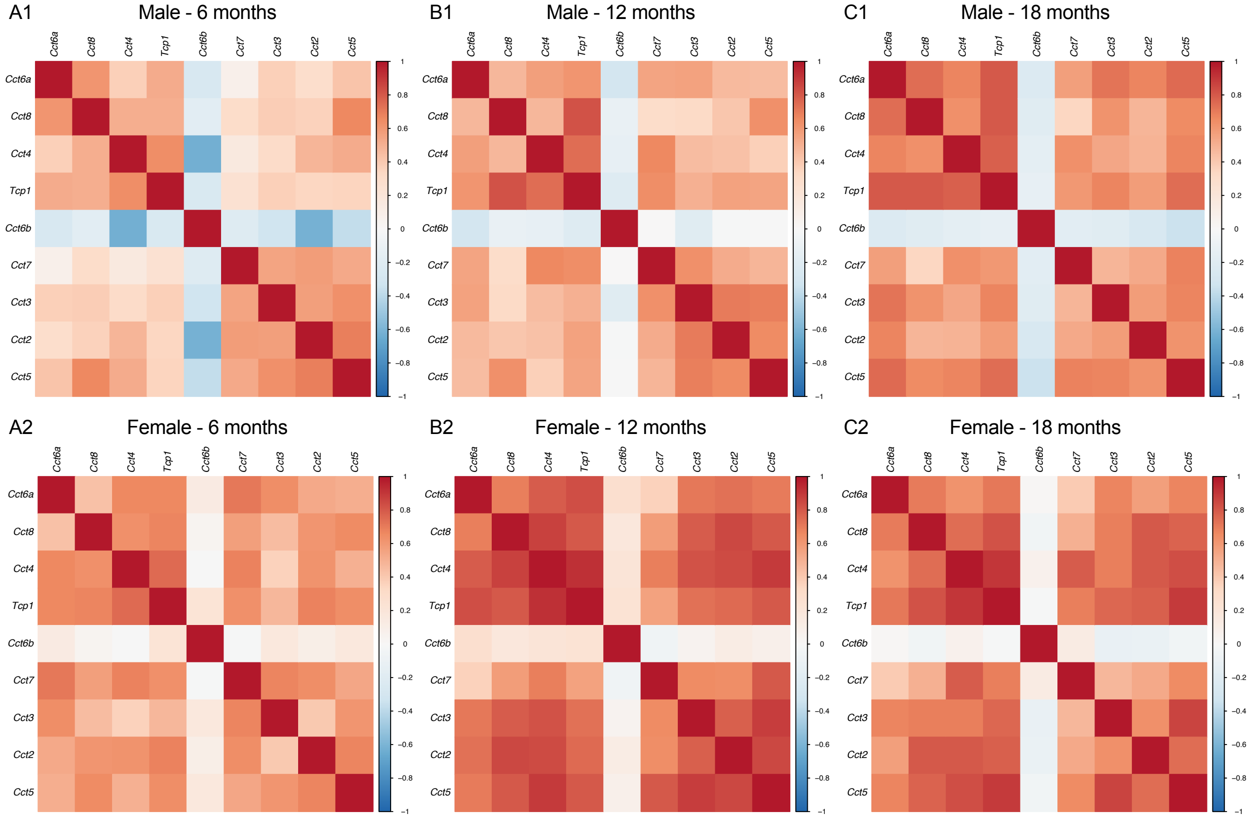

Cytoplasmic ribosomal large subunit

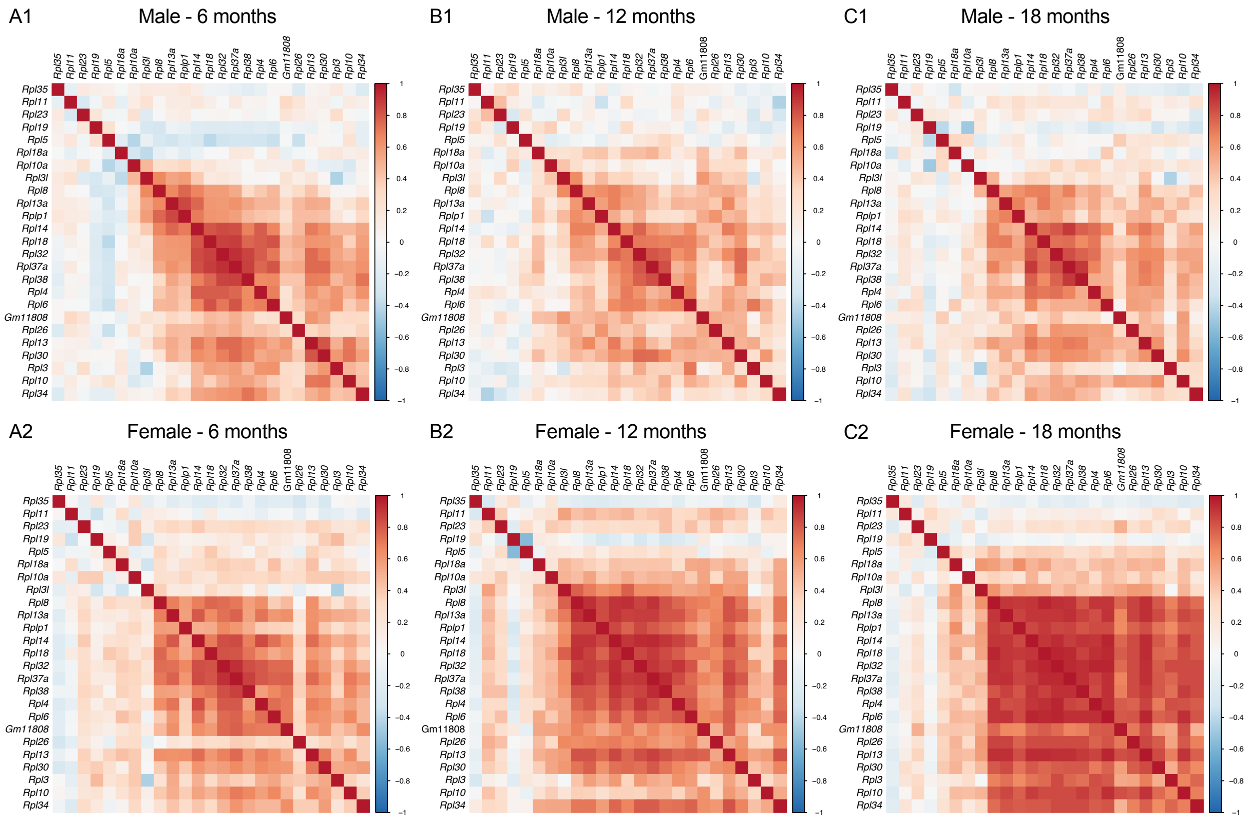

Large drosha complex

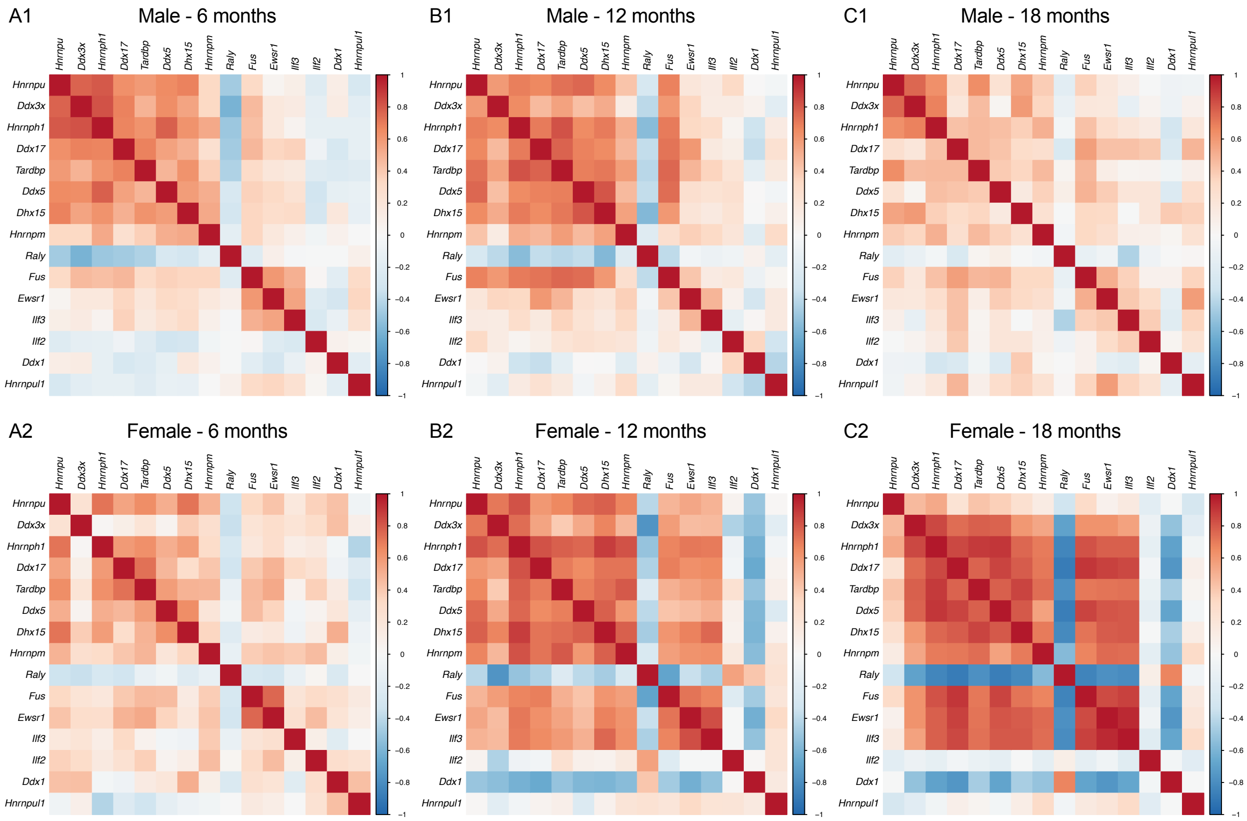

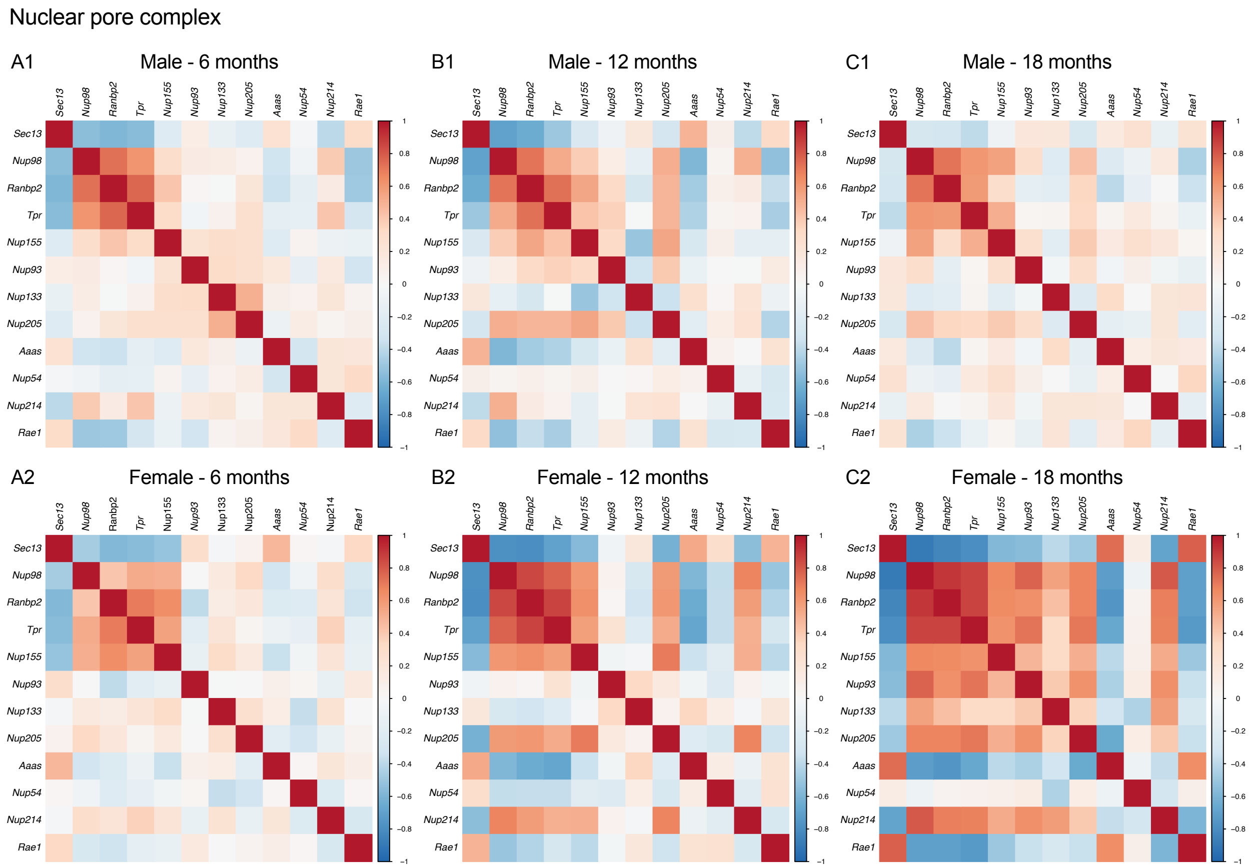

Supplemental Figure S5: Correlations for transcripts from protein complexes, stratified by age and sex. Heatmaps represent the correlation coefficients for transcript expression between members of the protein complexes that were most affected by age (26S proteasome, chaperonin-containing T complex, cytoplasmic ribosomal large subunit, large drosha complex and nuclear pore complex). Each column represents a heatmap for the age groups of 6 (A), 12 (B) and 18 months (C). The top row (1) represents males, and the bottom row (2) represents females.

26S proteasome

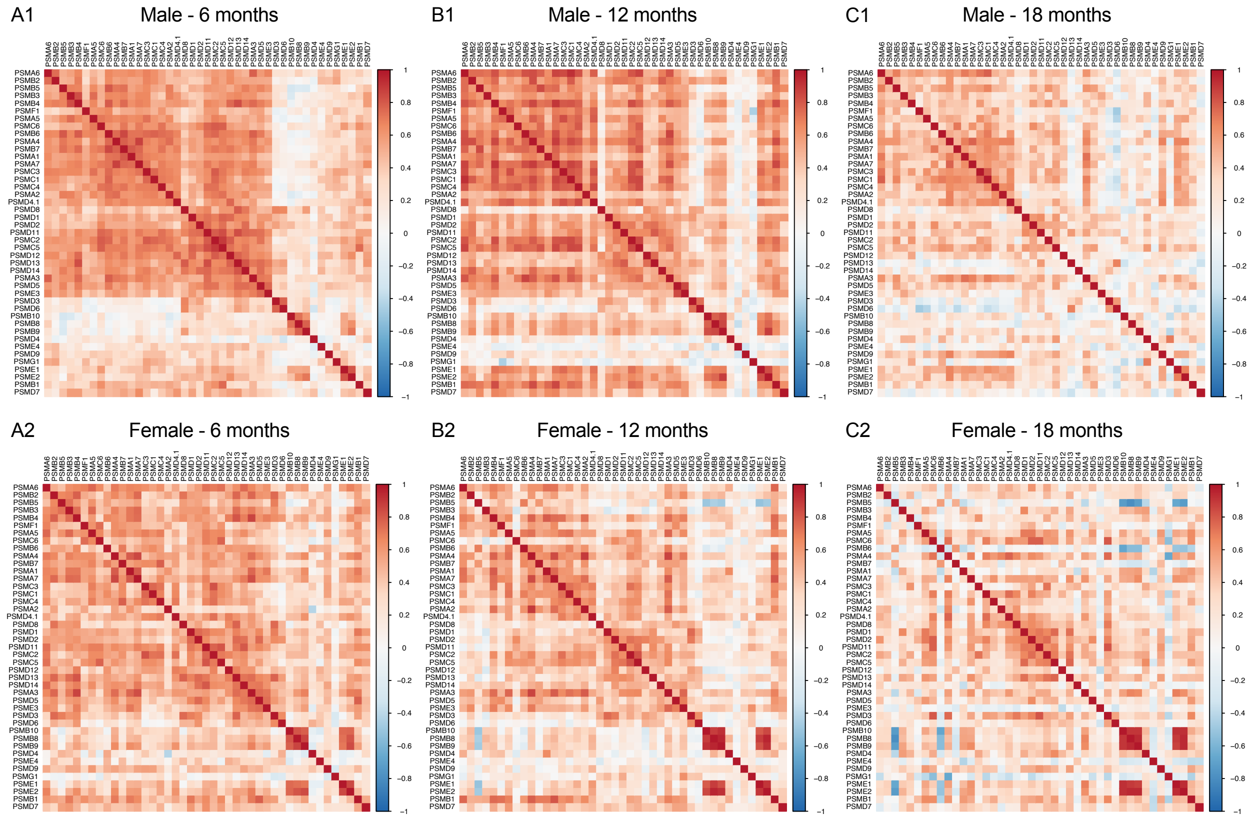

Chaperonin-containing T complex

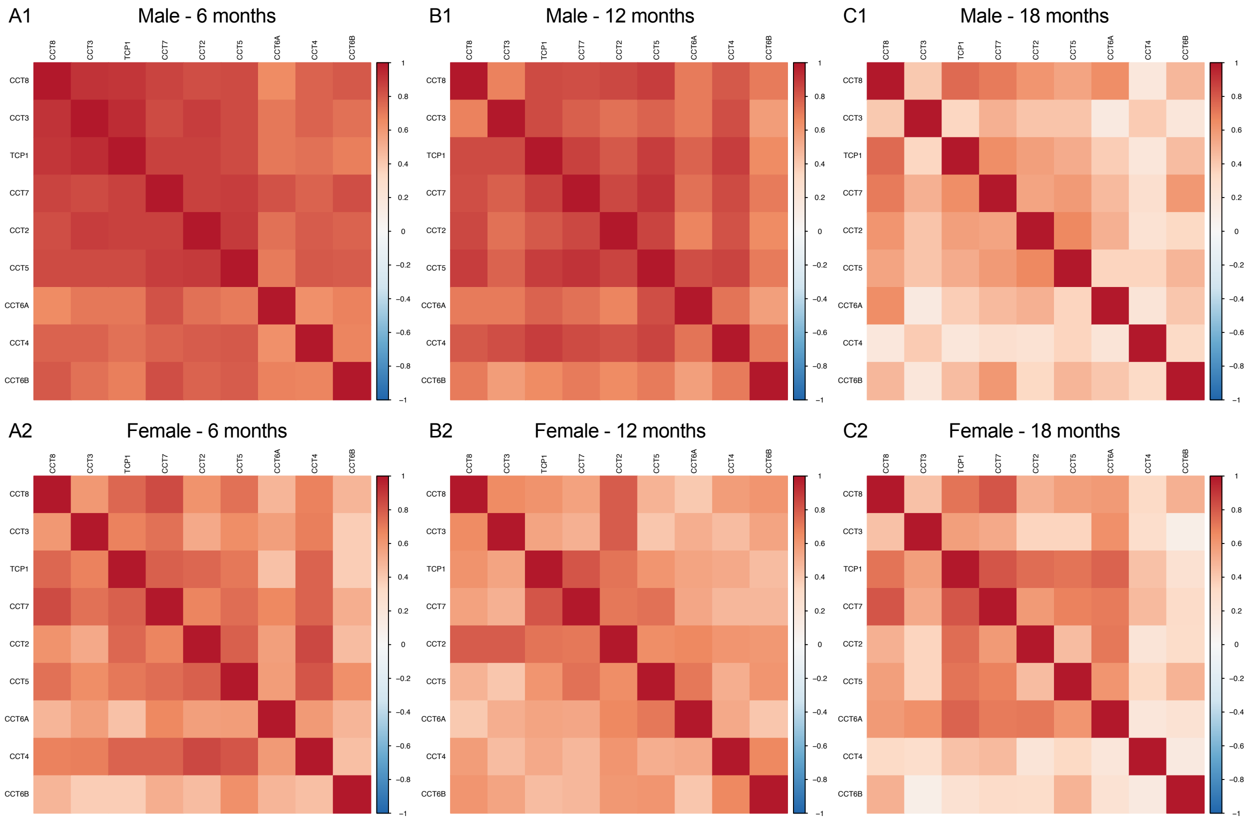

Cytoplasmic ribosomal large subunit

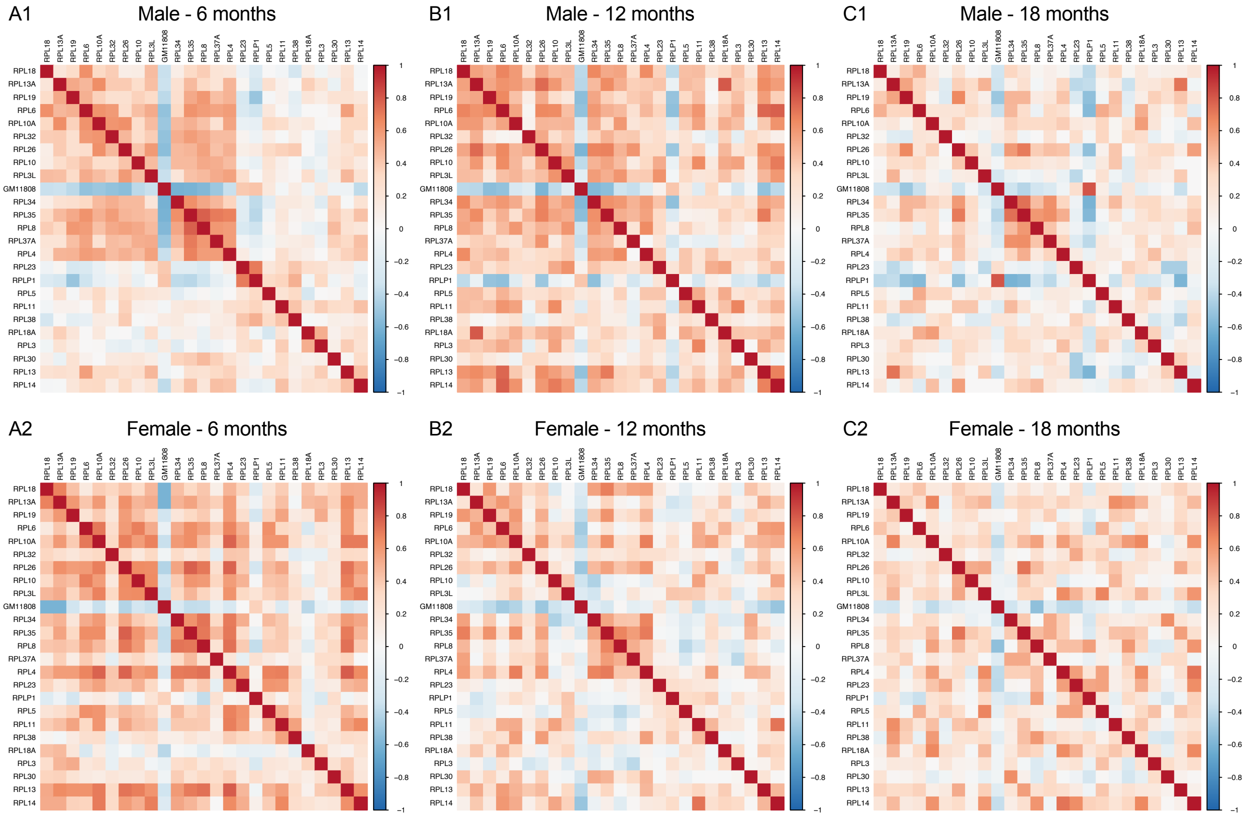

Large drosha complex

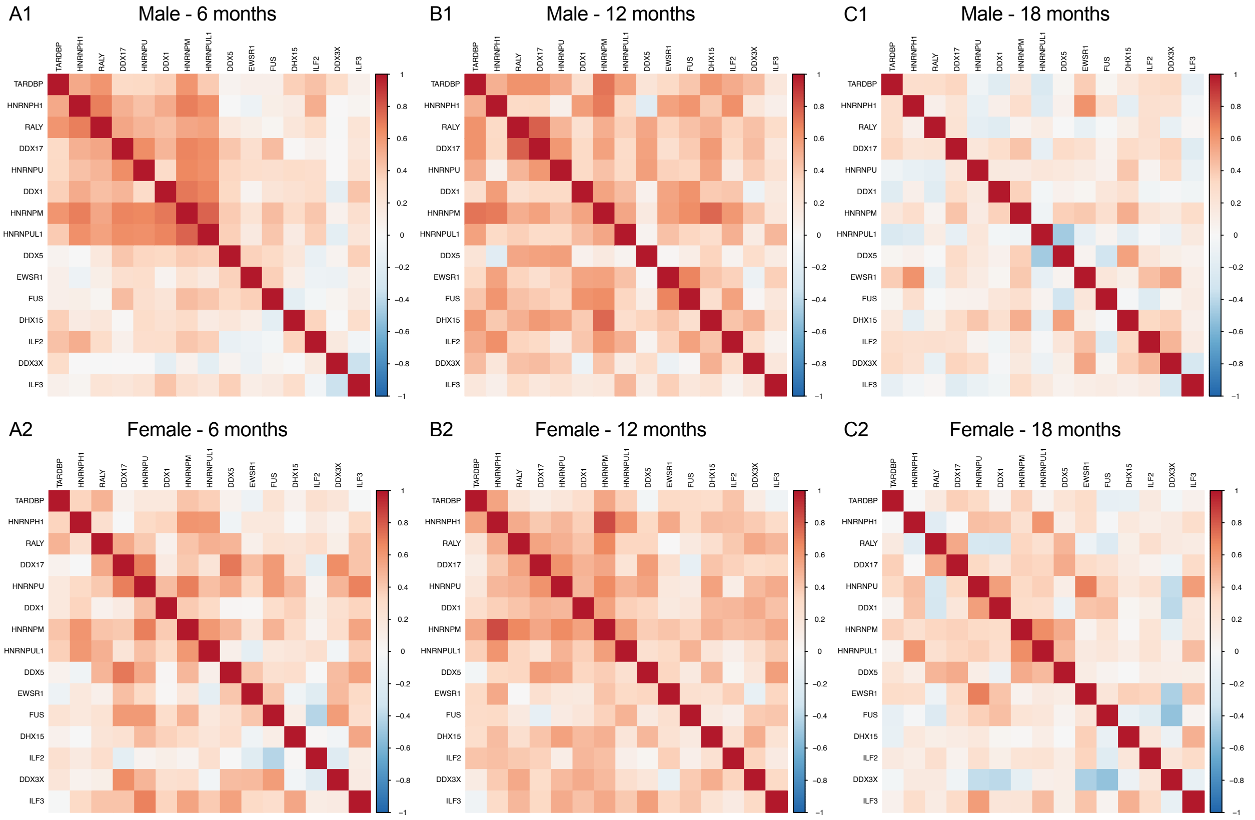

Nuclear pore complex

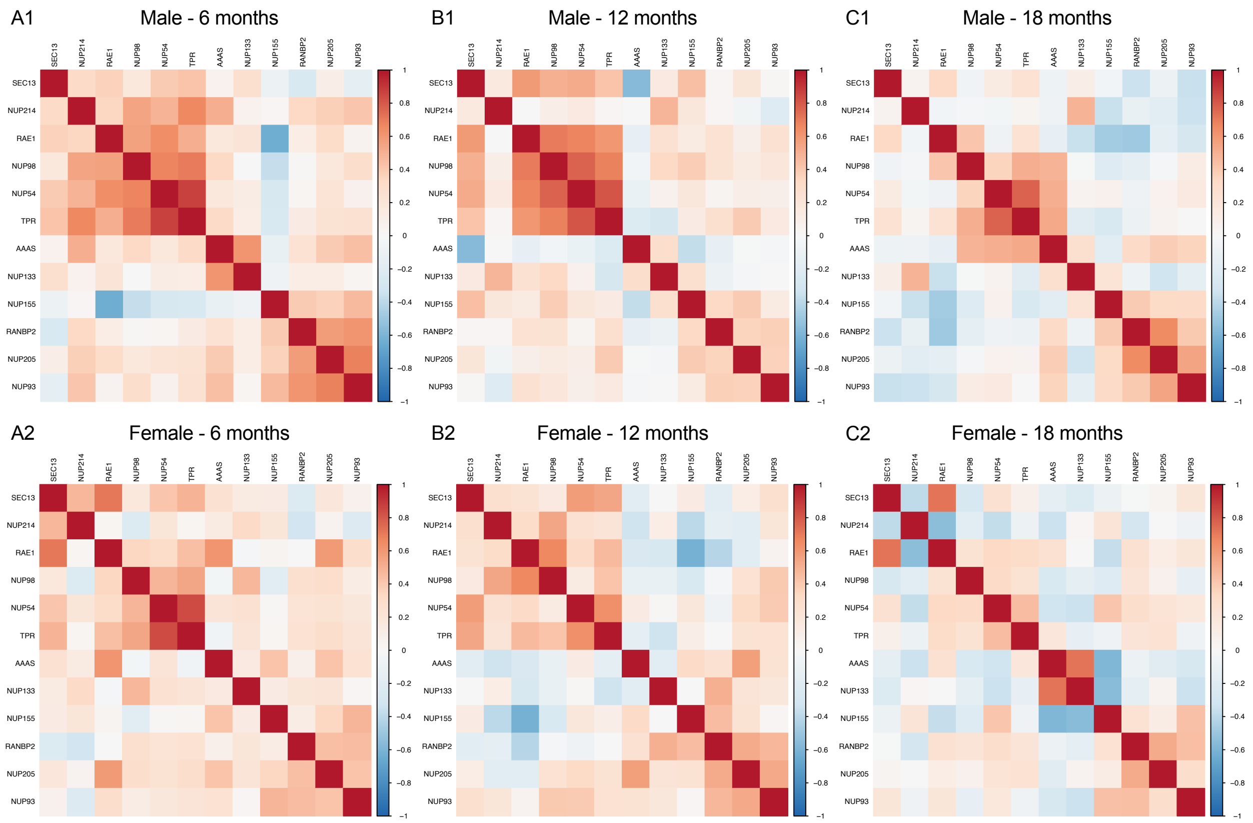

Supplemental Figure S6: Correlations for proteins from protein complexes, stratified by age and sex. Heatmaps represent the correlation coefficients for protein abundance between members of the protein complexes that were most affected by age (26S proteasome, chaperonin-containing T complex, cytoplasmic ribosomal large subunit, large drosha complex and nuclear pore complex). Each column represents a heatmap for the age groups of 6 (A), 12 (B) and 18 months (C). The top row (1) represents males, and the bottom row (2) represents females.

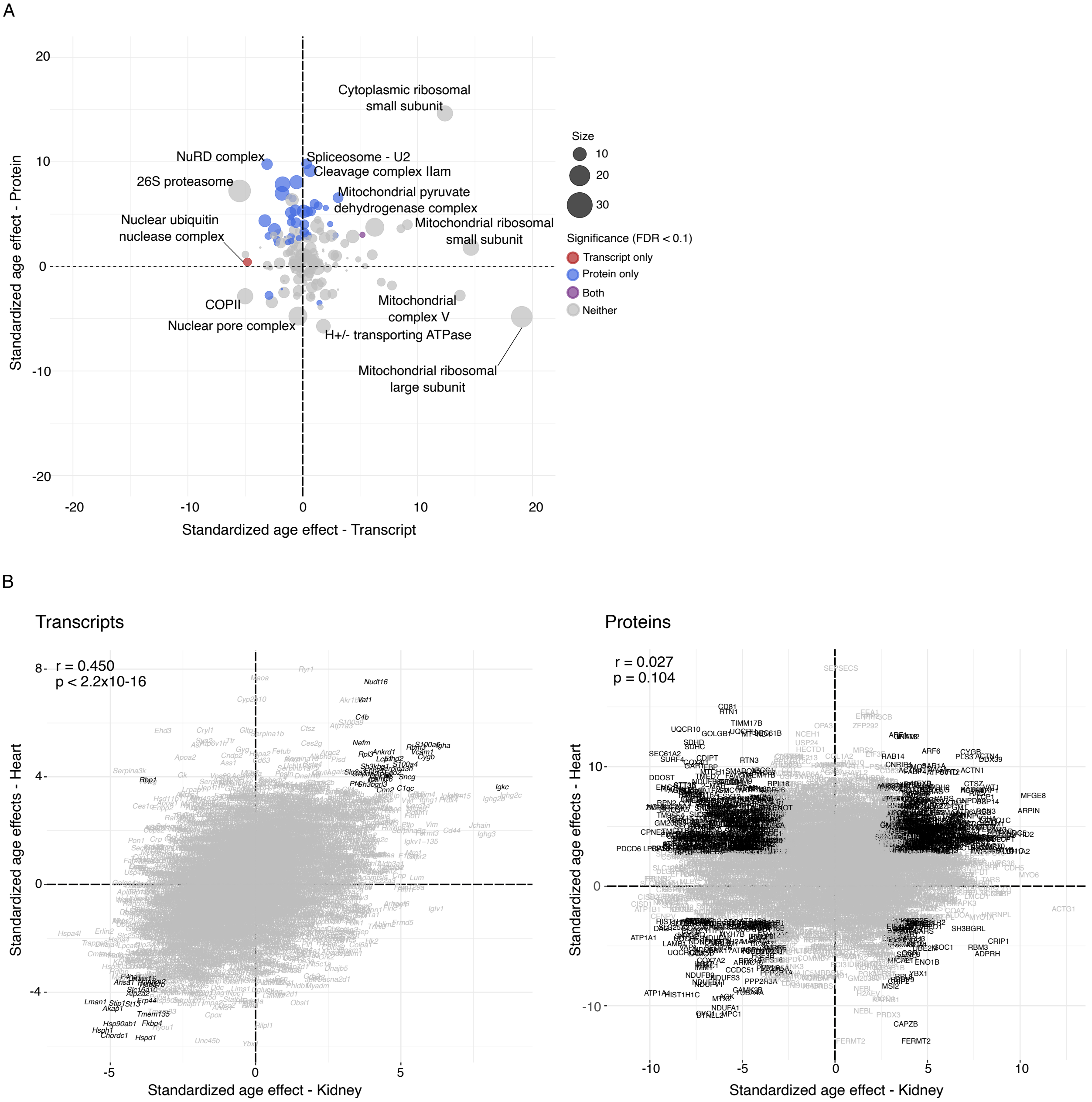

Supplemental Figure S7: Comparison of age effects between heart and kidney tissues. A) Standardized age effects on protein complex correlation in the kidney on proteins (y-axis) by transcripts (x-axis). Protein complex correlations mostly increase with age for proteins in kidney, unlike in the heart where they decrease (Figure 4A). Red points indicate a significant (FDR < 0.1) age effect for transcripts only, blue points for proteins only, purple points for both transcripts and proteins, and grey points indicate non-significant age effects for either. Size of points represents the total number of subunits in the complex for which we have data. Horizontal and vertical lines at 0 included for reference. B) Comparisons of standardized age effects between kidney (x-axis) and heart (y-axis) for transcripts (left) and proteins (right) reveal that there is reduced correlation between the tissues for proteins compared to transcripts. Age effects are reported as change in expression or abundance per year. Black labels represent genes with significant (FDR > 0.01) age-related changes for transcript and protein in both tissues.

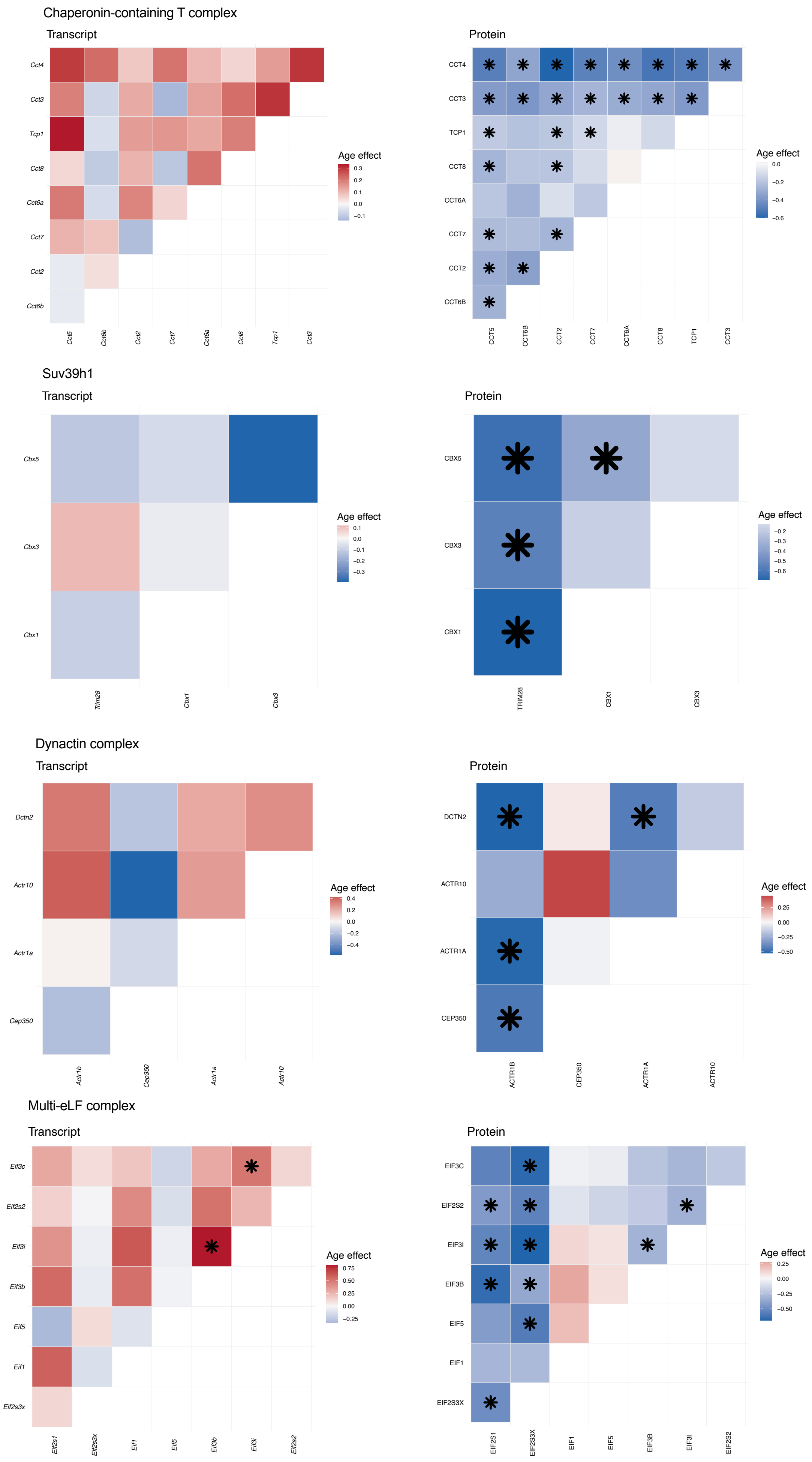

Chaperonin-containing T complex
Transcript
Cct4
Cct3
Tcp1
Cct8
Cct6a
Cct7
Cct2
Cct6b
Cct5
Cct6b
Cct2
Cct7
Cct6a
Cct8
Tcp1
Cct3
Age effect
0.3
0.2
0.1
0.0
−0.1
Protein
CCT4
CCT3
TCP1
CCT8
CCT6A
CCT7
CCT2
CCT6B
CCT5
CCT6B
CCT2
CCT7
CCT6A
CCT8
TCP1
CCT3
Age effect
0.0
−0.2
−0.4
−0.6
Suv39h1
Transcript
Cbx5
Cbx3
Cbx1
Trim28
Cbx1
Cbx3
Age effect
0.1
0.0
−0.1
−0.2
−0.3
Protein
CBX5
CBX3
CBX1
TRIM28
CBX1
CBX3
Age effect
−0.2
−0.3
−0.4
−0.5
−0.6
Dynactin complex
Transcript
Dctn2
Actr10
Actr1a
Cep350
Actr1b
Cep350
Actr1a
Actr10
Age effect
0.4
0.2
0.0
−0.2
−0.4
Protein
DCTN2
ACTR10
ACTR1A
CEP350
ACTR1B
CEP350
ACTR1A
ACTR10
Age effect
0.25
0.00
−0.25
−0.50
Multi-eLF complex
Transcript
Eif3c
Eif2s2
Eif3i
Eif3b
Eif5
Eif1
Eif2s3x
Eif2s1
Eif2s3x
Eif1
Eif5
Eif3b
Eif3i
Eif2s2
Age effect
0.75
0.50
0.25
0.00
−0.25
Protein
EIF3C
EIF2S2
EIF3I
EIF3B
EIF5
EIF1
EIF2S3X
EIF2S1
EIF2S3X
EIF1
EIF5
EIF3B
EIF3I
EIF2S2
Age effect
0.25
0.00
−0.25
−0.50

Large drosha complex

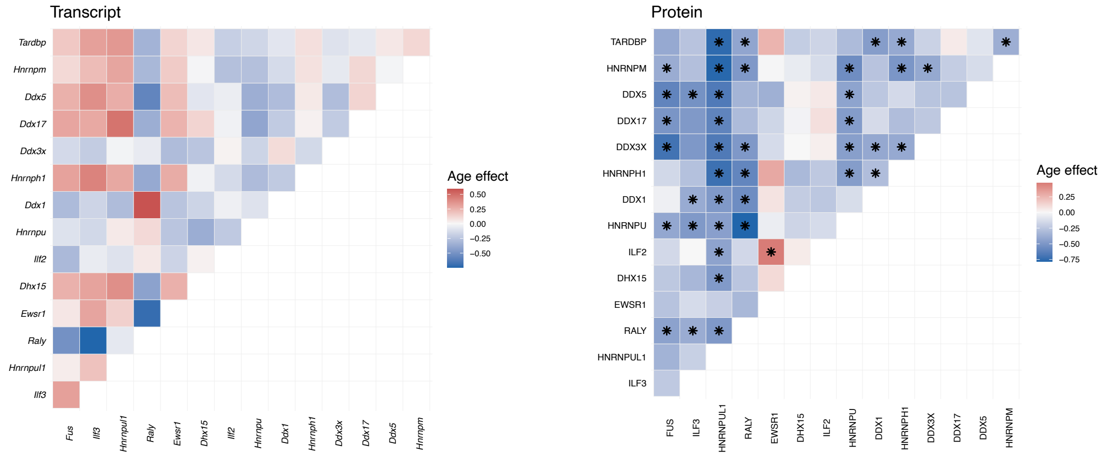

Ubiquilin-proteasome complex

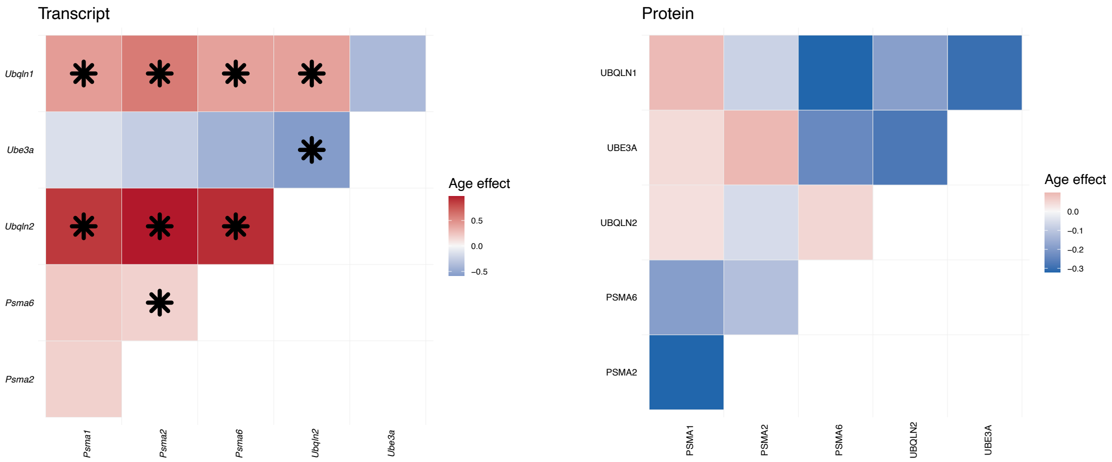

TNF-alpha/NF-kappa B signaling complex 7

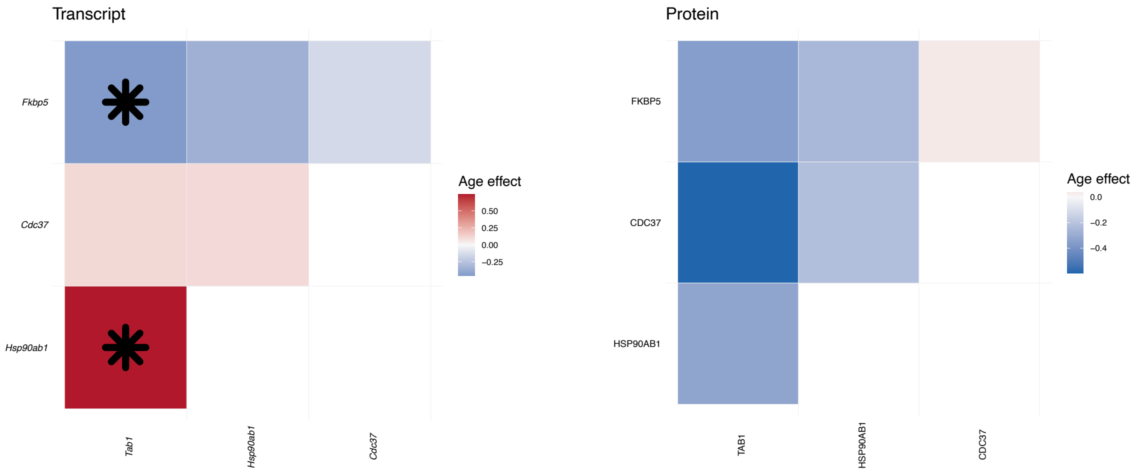

SMG-1-Upf1-eRF1-eRF3 (SURF) complex

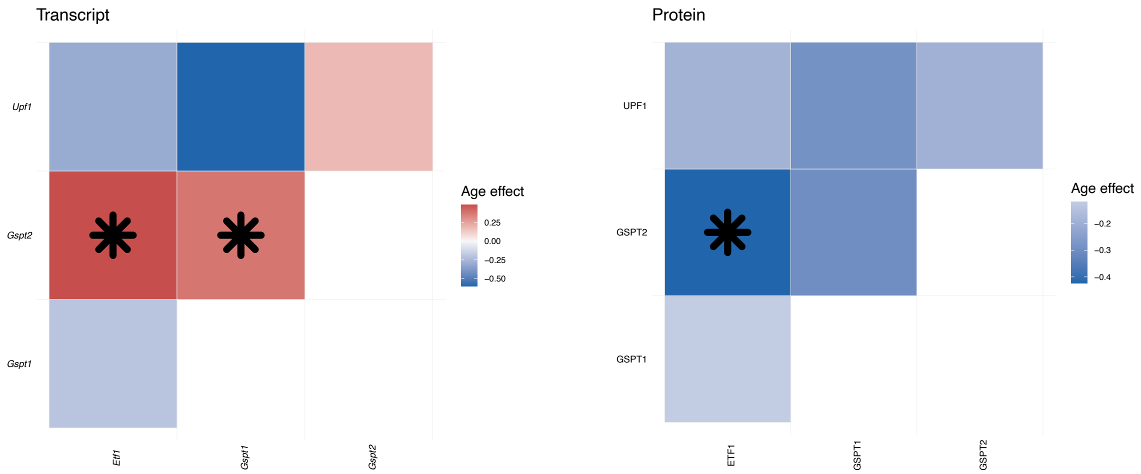

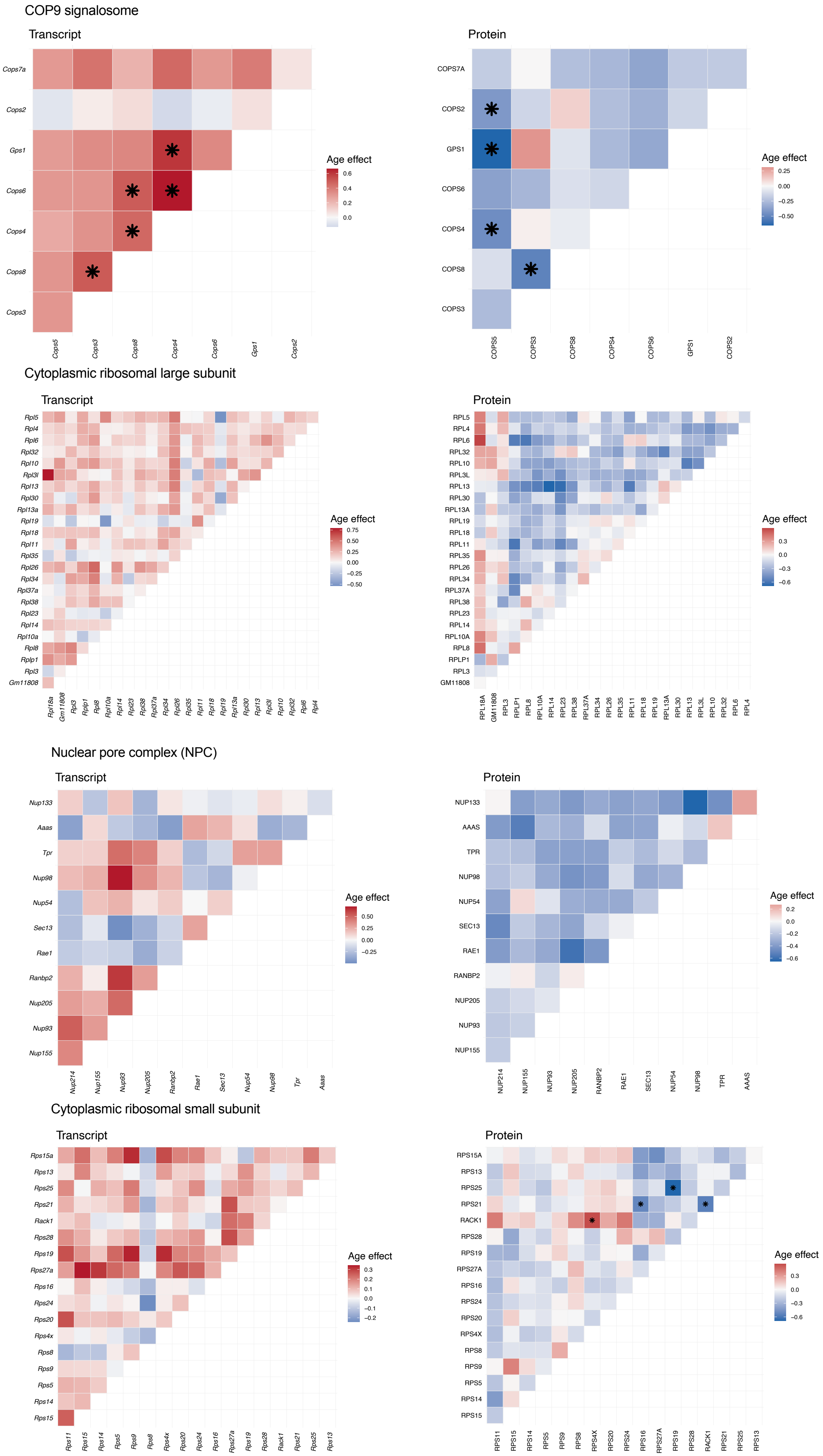
COP9 signalosome
Transcript
Cops7a Cops2 Gps1 Cops6 Cops4 Cops8 Cops3
Cops5 Cops3 Cops8 Cops4 Cops6 Gps1 Cops2
Age effect
0.6 0.4 0.2 0.0
Protein
COPS7A COPS2 GPS1 COPS6 COPS4 COPS8 COPS3
COPS5 COPS3 COPS8 COPS4 COPS6 GPS1 COPS2
Age effect
0.25 0.00 −0.25 −0.50
Cytoplasmic ribosomal large subunit
Transcript
Rpl5 Rpl4 Rpl6 Rpl32 Rpl10 Rpl3l Rpl13 Rpl30 Rpl13a Rpl19 Rpl18 Rpl11 Rpl35 Rpl26 Rpl34 Rpl37a Rpl38 Rpl23 Rpl14 Rpl10a Rpl8 Rplp1 Rpl3 Gm11808
Rpl18a Gm11808 Rpl3 Rplp1 Rpl8 Rpl10a Rpl14 Rpl23 Rpl38 Rpl37a Rpl34 Rpl26 Rpl35 Rpl11 Rpl18 Rpl19 Rpl13a Rpl30 Rpl13 Rpl3l Rpl10 Rpl32 Rpl6 Rpl4
Age effect
0.75 0.50 0.25 0.00 −0.25 −0.50
Protein
RPL5 RPL4 RPL6 RPL32 RPL10 RPL3L RPL13 RPL30 RPL13A RPL19 RPL18 RPL11 RPL35 RPL26 RPL34 RPL37A RPL38 RPL23 RPL14 RPL10A RPL8 RPLP1 RPL3 GM11808
RPL18A GM11808 RPL3 RPLP1 RPL8 RPL10A RPL14 RPL23 RPL38 RPL37A RPL34 RPL26 RPL35 RPL11 RPL18 RPL19 RPL13A RPL30 RPL13 RPL3L RPL10 RPL32 RPL6 RPL4
Age effect
0.3 0.0 −0.3 −0.6
Nuclear pore complex (NPC)
Transcript
Nup133 Aaas Tpr Nup98 Nup54 Sec13 Rae1 Ranbp2 Nup205 Nup93 Nup155
Nup214 Nup155 Nup93 Nup205 Ranbp2 Rae1 Sec13 Nup54 Nup98 Tpr Aaas
Age effect
0.50 0.25 0.00 −0.25
Protein
NUP133 AAAS TPR NUP98 NUP54 SEC13 RAE1 RANBP2 NUP205 NUP93 NUP155
NUP214 NUP155 NUP93 NUP205 RANBP2 RAE1 SEC13 NUP54 NUP98 TPR AAAS
Age effect
0.2 0.0 −0.2 −0.4 −0.6
Cytoplasmic ribosomal small subunit
Transcript
Rps15a Rps13 Rps25 Rps21 Rack1 Rps28 Rps19 Rps27a Rps16 Rps24 Rps20 Rps4x Rps8 Rps9 Rps5 Rps14 Rps15
Rps11 Rps15 Rps14 Rps5 Rps9 Rps8 Rps4x Rps20 Rps24 Rps16 Rps27a Rps19 Rps28 Rack1 Rps21 Rps25 Rps13
Age effect
0.3 0.2 0.1 0.0 −0.1 −0.2
Protein
RPS15A RPS13 RPS25 RPS21 RACK1 RPS28 RPS19 RPS27A RPS16 RPS24 RPS20 RPS4X RPS8 RPS9 RPS5 RPS14 RPS15
RPS11 RPS15 RPS14 RPS5 RPS9 RPS8 RPS4X RPS20 RPS24 RPS16 RPS27A RPS19 RPS28 RACK1 RPS21 RPS25 RPS13
Age effect
0.3 0.0 −0.3 −0.6

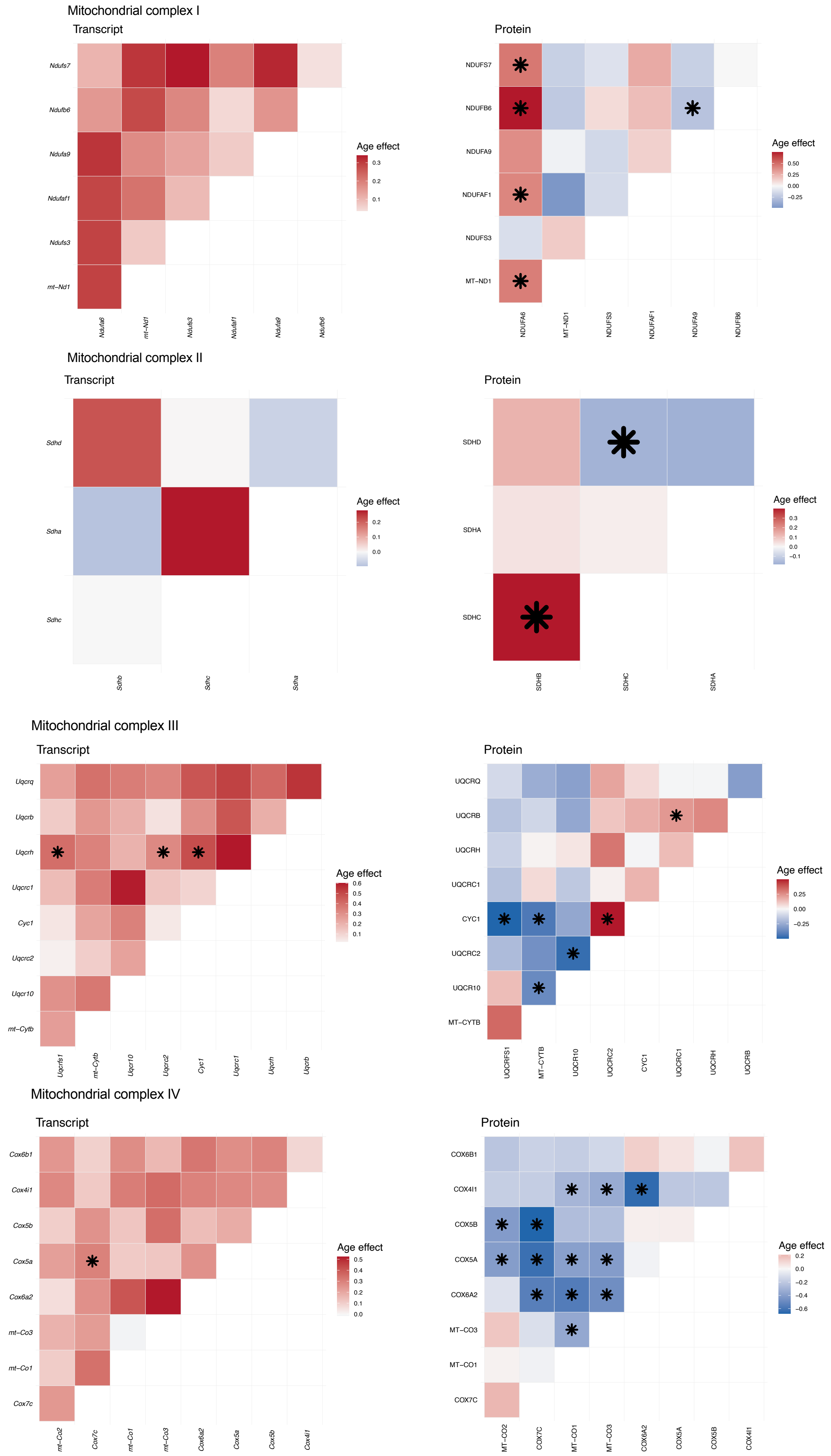

Mitochondrial complex I
Transcript
Ndufs7
Ndufb6
Ndufa9
Ndufaf1
Ndufs3
mt−Nd1
Ndufa6
mt−Nd1
Ndufs3
Ndufaf1
Ndufa9
Ndufb6
Age effect
0.3
0.2
0.1
Protein
NDUFS7
NDUFB6
NDUFA9
NDUFAF1
NDUFS3
MT−ND1
NDUFA6
MT−ND1
NDUFS3
NDUFAF1
NDUFA9
NDUFB6
Age effect
0.50
0.25
0.00
−0.25
Mitochondrial complex II
Transcript
Sdhd
Sdha
Sdhc
Sdhb
Sdhc
Sdha
Age effect
0.2
0.1
0.0
Protein
SDHD
SDHA
SDHC
SDHB
SDHC
SDHA
Age effect
0.3
0.2
0.1
0.0
−0.1
Mitochondrial complex III
Transcript
Uqcrq
Uqcrb
Uqcrh
Uqcrc1
Cyc1
Uqcrc2
Uqcr10
mt−Cytb
Uqcrfs1
mt−Cytb
Uqcr10
Uqcrc2
Cyc1
Uqcrc1
Uqcrh
Uqcrb
Age effect
0.6
0.5
0.4
0.3
0.2
0.1
Protein
UQCRQ
UQCRB
UQCRH
UQCRC1
CYC1
UQCRC2
UQCR10
MT−CYTB
UQCRFS1
MT−CYTB
UQCR10
UQCRC2
CYC1
UQCRC1
UQCRH
UQCRB
Age effect
0.25
0.00
−0.25
Mitochondrial complex IV
Transcript
Cox6b1
Cox4i1
Cox5b
Cox5a
Cox6a2
mt−Co3
mt−Co1
Cox7c
mt−Co2
Cox7c
mt−Co1
mt−Co3
Cox6a2
Cox5a
Cox5b
Cox4i1
Age effect
0.5
0.4
0.3
0.2
0.1
0.0
Protein
COX6B1
COX4I1
COX5B
COX5A
COX6A2
MT−CO3
MT−CO1
COX7C
MT−CO2
COX7C
MT−CO1
MT−CO3
COX6A2
COX5A
COX5B
COX4I1
Age effect
0.2
0.0
−0.2
−0.4
−0.6

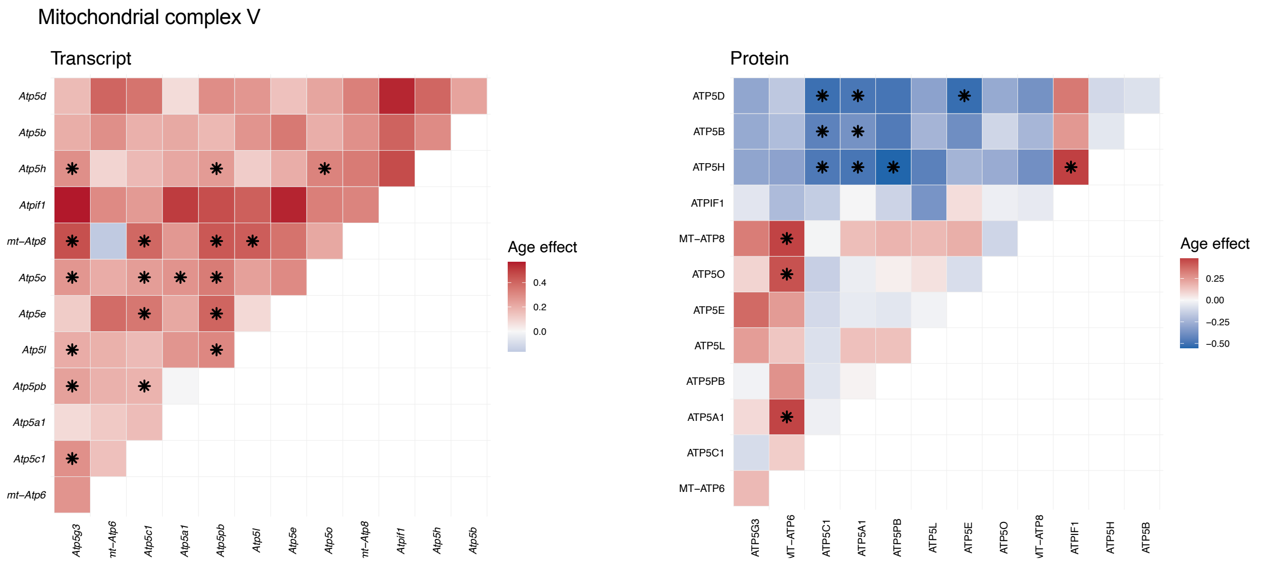

Supplemental Figure S8: Age effects on protein complex correlations. Heatmaps represent the age-related change in correlations between gene pairs for members of 17 protein complexes (Chaperonin-containing T complex, Suv39h1, Dynactin complex, Multi-eLF complex, Large drosha complex, Ubiquilin-proteasome complex, TNF-alpha/NF-kappa B signaling complex 7, SURF complex, COP9 signalosome, Cytoplasmic ribosomal large subunit, NPC, Cytoplasmic ribosomal small subunit, and mitochondrial respiratory chain complexes I-V). Age effects on transcripts are shown on the left and age effects on proteins are shown on the right. These protein complexes were detected by either having a strong overall age effect or by presenting a high proportion of pairwise correlations with significant changes due to age. Asterisks highlight gene pairs with significant (FDR < 0.1) changes in correlation.

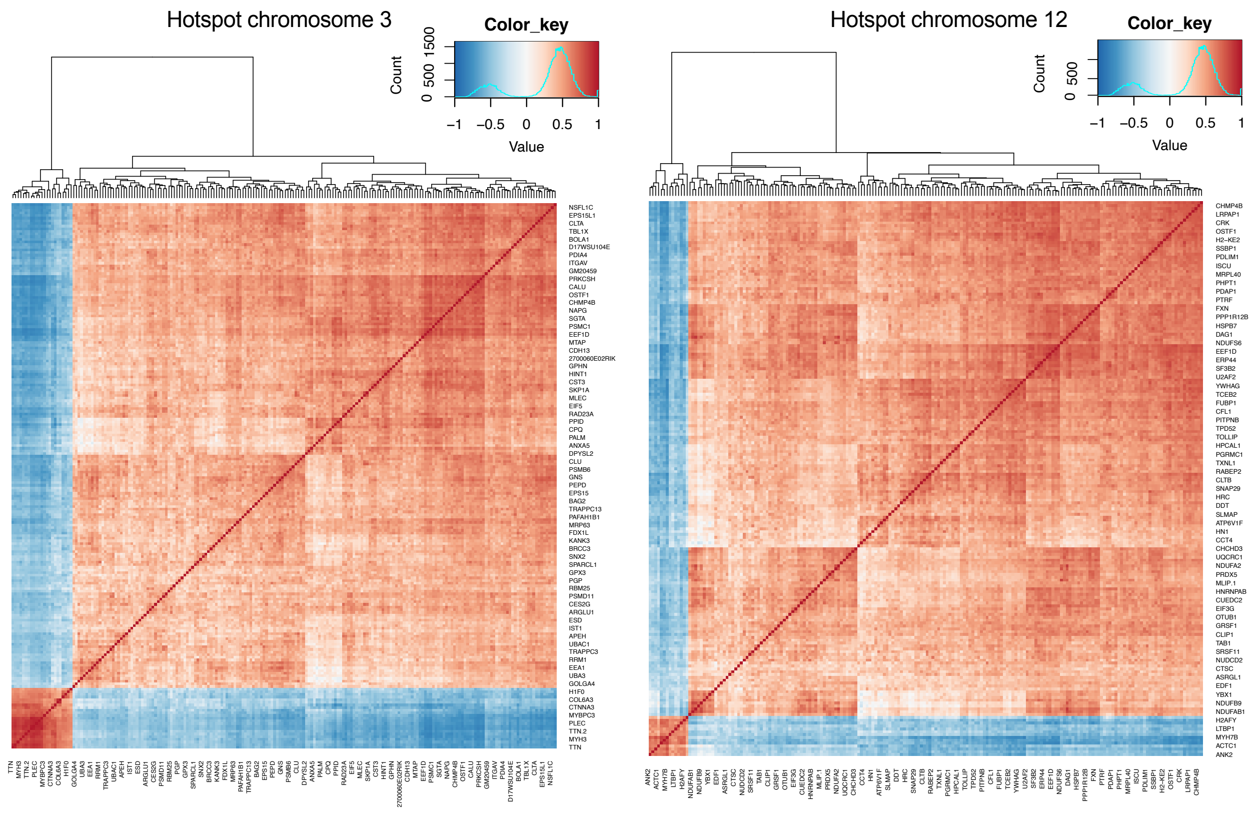

Supplemental Figure S9: Correlation between proteins that mapped to age-pQTL hotspots. Heatmaps represent the correlation coefficients between proteins that mapped to age-pQTL hotspots regions on chromosomes 3 (left) and chromosome 12 (right), after filtering out lowly correlated members (|r| < 0.3). The hotspots consist of tightly (positively and negatively) correlated groups of proteins that potentially share common genetic drivers.
