# Supplementary material for: Genome-wide transcript and protein analysis highlights the role of protein homeostasis in the aging mouse heart
Source: Genome Res. 2022 May;32(5):838–52. doi: 10.1101/gr.275672.121 (PMC9104701; doi:10.1101/gr.275672.121)
Supplement: Supplemental Material [file supp_gr.275672.121_Supplemental_Scripts.zip › Scripts/HeartWorkflow.pdf]

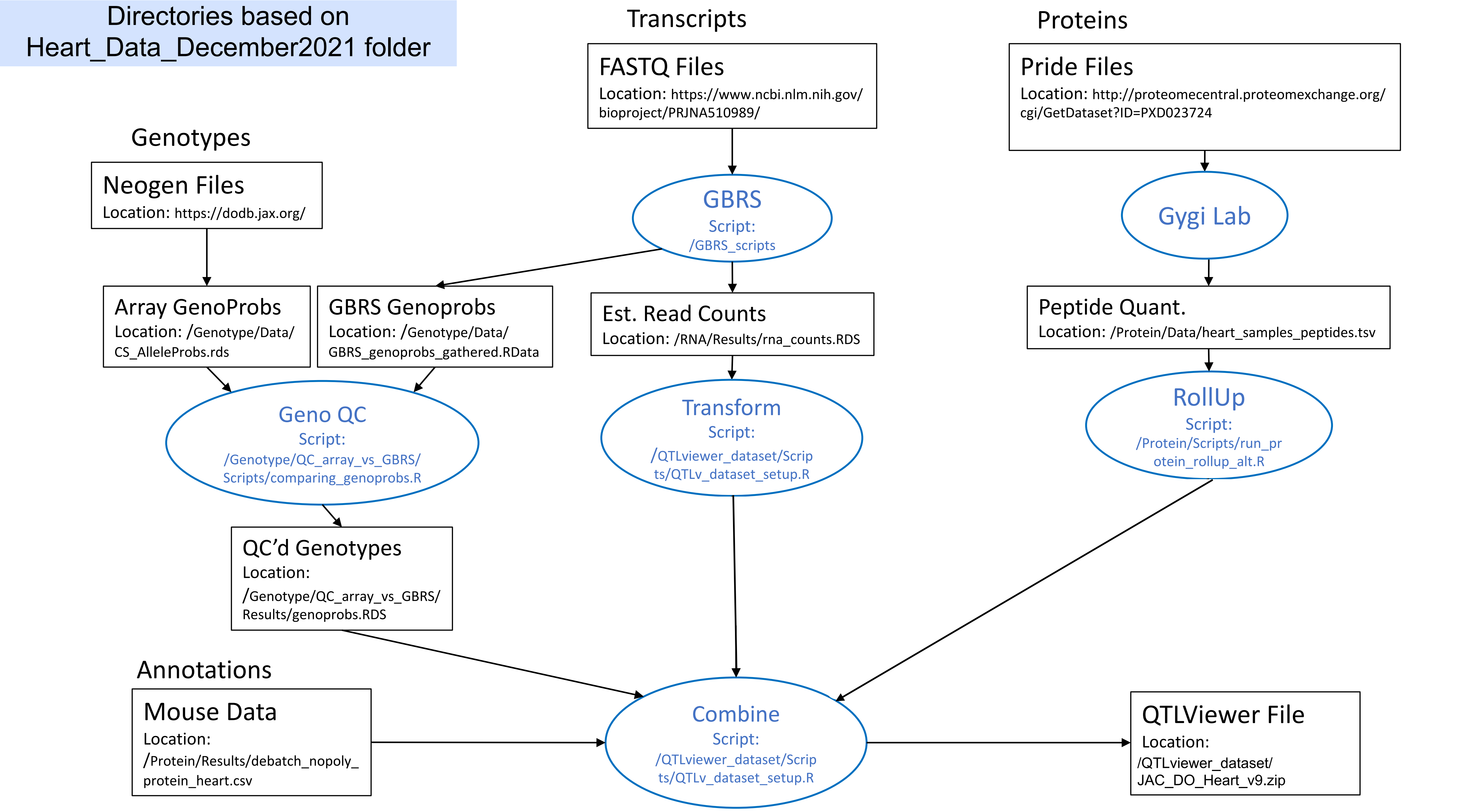
Directories based on Heart_Data_December2021 folder
Transcripts
FASTQ Files
Location: https://www.ncbi.nlm.nih.gov/bioproject/PRJNA510989/
GBRS
Script: /GBRS_scripts
Est. Read Counts
Location: /RNA/Results/rna_counts.RDS
Transform
Script: /QTLviewer_dataset/Scripts/QTLv_dataset_setup.R
Proteins
Pride Files
Location: http://proteomecentral.proteomexchange.org/cgi/GetDataset?ID=PXD023724
Gygi Lab
Peptide Quant.
Location: /Protein/Data/heart_samples_peptides.tsv
RollUp
Script: /Protein/Scripts/run_protein_rollup_alt.R
Genotypes
Neogen Files
Location: https://dodb.jax.org/
Array GenoProbs
Location: /Genotype/Data/CS_AlleleProbs.rds
GBRS Genoprobs
Location: /Genotype/Data/GBRS_genoprobs_gathered.RData
Geno QC
Script: /Genotype/QC_array_vs_GBRS/Scripts/comparing_genoprobs.R
QC'd Genotypes
Location: /Genotype/QC_array_vs_GBRS/Results/genoprobs.RDS
Annotations
Mouse Data
Location: /Protein/Results/debatch_nopoly_protein_heart.csv
Combine
Script: /QTLviewer_dataset/Scripts/QTLv_dataset_setup.R
QTLViewer File
Location: /QTLviewer_dataset/JAC_DO_Heart_v9.zip
